# Subtilisin-like Pr1 proteases marking the evolution of pathogenicity in a wide-spectrum insect-pathogenic fungus

**DOI:** 10.1080/21505594.2020.1749487

**Published:** 2020-04-23

**Authors:** Ben-Jie Gao, Ya-Ni Mou, Sen-Miao Tong, Sheng-Hua Ying, Ming-Guang Feng

**Affiliations:** a MOE Laboratory of Biosystems Homeostasis & Protection, Institute of Microbiology, College of Life Sciences, Zhejiang University, Hangzhou, Zhejiang, China; b College of Agricultural and Food Science, Zhejiang A and F University, Lin’an, Zhejiang, China

**Keywords:** Entomopathogenic fungi, subtilisin-like Pr1 proteases, extracellular Pr1 activity, insect cuticle degradation, pathogenicity, virulence

## Abstract

Subtilisin-like Pr1 proteases of insect-pathogenic fungi are a large family of extracellular cuticle-degrading enzymes that presumably determine a capability of hyphal invasion into insect hemocoel through normal cuticle infection, but remain poorly understood although often considered as virulence factors for genetic improvement of fungal potential against pests. Here, we report that not all of 11 Pr1 family members necessarily function in *Beauveria bassiana*, an ancient wide-spectrum pathogen evolved insect pathogenicity ~200 million years ago. These Pr1 proteases are phylogenetically similar to or distinct from 11 homologues (Pr1A–K) early named in *Metarhizium anisopliae* complex, a young entomopathogen lineage undergoing molecular evolution toward Pr1 diversification, and hence renamed Pr1A1/A2, Pr1B1–B3, Pr1 C, Pr1F1–F4,4 and Pr1 G, respectively. Multiple analyses of all single gene-deleted and rescued mutants led to the recognition of five conserved members (Pr1C, Pr1G, Pr1A2, Pr1B1, and Pr1B2) contributing significantly to the fungal pathogenicity to insect. The conserved Pr1 proteases were proven to function only in cuticle degradation, individually contribute 19–29% to virulence, but play no role in post-infection cellular events critical for fungal killing action. Six other Pr1 proteases were not functional at all in either cuticle degradation during host infection or virulence-related cellular events post-infection. Therefore, only the five conserved proteases are collectively required for, and hence mark evolution of, insect pathogenicity in *B. bassiana*. These findings provide the first referable base for insight into the evolution of Pr1 family members in different lineages of fungal insect pathogens.

## Introduction

*Beauveria bassiana* and *Metarhizium anisopliae* complex are two representative lineages of filamentous fungal insect pathogens that serve as biological control agents of arthropod pests and main sources of fungal insecticides and acaricides (reviewed in [1,2]). These insect pathogens infect a host through the normal route of cuticular penetration by the hyphae from germ tubes of conidia attached to insect surface. Successful host infection relies on the activities of extracellular (proteolytic, chitinolytic, and lipolytic) enzymes secreted for cuticle degradation (reviewed in [3–5]) and selectively on the mechanistic pressure of specialized hyphal structures, such as appressoria often considered to be critical for host infection by *Metarhizium* species [6,7] but rarely formed in the infection course of *B. bassiana*. Among the enzymes secreted during fungal invasion, subtilisin-like Pr1 proteases have drawn extensive attention in the past decades due to their presumable roles in determining fungal ability to cause host mycosis and death [3–5,8]. Early efforts resulted in one or two *pr1* genes cloned from *M. anisopliae* [9,10] and *B. bassiana* [11,12]. The *M. anisopliae* strains engineered for overexpression of cloned *pr1A* gene showed an enhanced virulence [13]. This study established an evidence for a Pr1 protease to be considered as a key virulence factor [6,12,14,15], and has promoted attempts to enhance fungal virulence by making use of a *pr1* gene alone or together with a chitinase gene [16–19] or bacterial toxin gene [20]. Extracellular Pr1 activity (EPA) required for host cuticle degradation is often used as a biochemical marker of fungal virulence against target pest species [21,22].

After the discovery of a couple of *pr1* genes in the early studies, 11 proteases constituting a large Pr1 family were found in *M. anisopliae* strains through the analyses of expressed sequence tag (EST) libraries and named Pr1A–K, respectively, [15]. The Pr1 family members are considered to be functionally non-redundant for either scavenging for nutrients or insect pathogenicity, and phylogenetically fall into two classes, namely class I (“bacterial”) subtilisin (Pr1C only) and class II proteinase K-like subtilisins, which are further classified to extracellular subfamily 1 (SF1; Pr1A, Pr1B, Pr1G, Pr1I, and Pr1K), extracellular subfamily 2 (SF2; Pr1D, Pr1E, Pr1F, and Pr1J) and endocellular subfamily 3 (SF3; Pr1H only) [23]. The nomenclature of all Pr1 proteases identified from the EST libraries is undoubtedly influential on the annotation of their homologues in later sequenced genomes of insect-pathogenic fungi, including *Metarhizium robertsii* and *M. acridum* [24] separated from *M. anisopliae* complex [25] and other distinct lineages, such as *Aschersonia, Beauveria, Cordyceps, Isaria, Lecanicillium* and *Nomuraea* [26–28]. These fungal genomes contain similar numbers of subtilisin-like Pr1 proteases that are phylogenetically close to or distinct from one another and those homologues named earlier. These hints at more or less divergence of some Pr1 family members in the evolution of fungal insect pathogenicity and a need for revision of their nomenclature or annotation through functional analysis. As far as known to date, only one or two *pr1* genes (*pr1A/B*) in *M. anisopliae* and *B. bassiana* have been functionally explored with the strains in which a *pr1* gene was overexpressed (13, 17–19) or disrupted [14]. However, most of the Pr1 family members have not been characterized yet, leaving it unknown whether and how they are involved in the cuticle degradation required for insect pathogenicity and contribute to fungal virulence in different lineages of fungal insect pathogens.

Fungal insect pathogenicity is a qualitative or all-or-none response of insect host to fungal invasion and conceptually distinct from fungal virulence, which is a quantitative or measurable characteristic of fungal ability to cause host mycosis and death (particularly important for killing action of formulated fungal cells against arthropod pests). It is necessary to distinguish the two concepts based on pathogenicity and virulence of microbial pathogens previously defined in a general sense [29]. Indeed, fungal virulence depends on not only a successful invasion by hyphal penetration through insect cuticle but also an array of cellular processes and events, which take place after fungal entry into host hemocoel and are regulated by complicated signaling pathways [30,31]. Upon entry into host hemocoel, for instance, penetrating hyphae turn into unicellular blastospores, namely hyphal bodies, to accelerate host death from mummification by yeast-like budding [32]. Near the time of host death, hyphal bodies turn back into hyphae to penetrate the cuticle again for outgrowth required for ultimate conidiation on the surfaces of insect cadavers. This dimorphic transition process is critical for fungal virulence, survival, and dispersal and has been shown to be controlled by the key activators of central development pathway in *B. bassiana* [33]. In addition, hyphal penetration through host cuticle and subsequent intrahaemocoel propagation leading to host mummification rely upon several families of antioxidiant enzymes to overcome oxidative stress of reactive oxygen species generated from host immunity defense [34]. Insect host also produces serine protease inhibitors in response to the fungal infection [35]. A successful infection depends on the fungal ability to rapidly sense the presence of the host immunity-derived protease inhibitors and antifungal peptides and to responsively synthesize chymotrypsin-like proteinases and metalloproteinases that enable to degrade the antifungal inhibitor molecules [36]. However, it is unclear whether, aside from a possible role in cuticle degradation, the Pr1 family members are involved in other important cellular events to determine the speed of host death, which is crucial for a virulence factor of fungal cells formulated for pest control.

Polyphylogenomics analyses [24,26,27] have revealed that fungal insect pathogenicity could have evolved from plant entophytes or pathogens around the Triassic-Jurassic boundary ~200 million years ago on the earth and that the *Beauveria*/*Cordyceps* lineage could have gained insect pathogenicity at least 130 million years earlier than the *Metarhizium* lineage well represented by *M. anisopliae* complex, which virtually includes multiple species to have been recognized through multigene phylogenetic analysis, such as the generalist *M. robertsii* and the specialist *M. acridum* [25]. The distinct histories of the two lineages in the evolution of insect pathogenicity hint at a possibility that the Pr1 proteases critical for host cuticle degradation in the infection course could be more conserved in *B. bassiana* than in *M. anisopliae* complex, in which the Pr1 family is being diversified through molecular evolution [37]. Among all insect pathogens known to date, *B. bassiana* has the broadest host spectrum covering almost all insect orders and Acari and serves as a main source of wide-spectrum fungal pesticides (reviewed in [1,2,38]). Interestingly, the annotated *B. bassiana* genome [27] comprises the same number of Pr1 proteases as does the *M. robertsii* genome [24] or revealed early in the EST libraries of *M. anisopliae* complex [15] but no endocellular SF3 member. This study sought to characterize biological functions of all *B. bassiana* Pr1 proteases by multiple analyses of single gene-deleted and rescued mutants, clarify which Pr1 proteases function in host infection and hence are essential for insect pathogenicity, identify conserved molecular marks of insect pathogenicity from the Pr1 family, and understand a significance of the conserved marks for the adaptation of *B. bassiana* to the broadest host spectrum. As presented below, class I Pr1C and four class II SF1 members are essential for total EPA and cuticular penetration required for insect pathogenicity and infection cycle but have no role in the virulence-related cellular events post-infection whereas two SF1 and four SF2 members are functionally redundant. These findings unveil a collective significance of the five recognized Pr1 proteases for both the insect pathogenicity and the broadest host spectrum of *B. bassiana* and establish a referable base for insight into molecular evolution of insect pathogenicity in different lineages of fungal insect pathogens.

## Results

### Phylogenetic ties of Pr1 proteases between Beauveria and Metarhizium lineages

Ten of 11 Pr1 proteases in the *B. bassiana* genome [27] were annotated as subtilisin-like proteases (Pr1 plus an uppercase letter or not) or subtilisin-like serine protease (only Pr1 C) in a fashion obviously following the earlier nomenclature of their homologues in *M. anisopliae* complex [15,23]. Another Pr1 was annotated as cuticle-degrading protease bassiasin I precursor, which is identical to CDEP1 reported previously [16,18]. In phylogeny, all *B. bassiana* Pr1 proteases fall into the clades of *Metarhizium* classes I (Pr1 C) and II (SF1 and SF2), but some SF1/2 members are distinct, at or below subclade levels, from those homologues early named in *M. anisopliae* (15,23) or annotated in *M. robertsii* [24], as illustrated in Supplementary Fig. S1. Particularly, 11 Pr1 proteases in *M. anisopliae* and *M. robertsii* are all identical in both phylogeny and nomenclature, and no homologue of SF3 Pr1 H exists in *B. bassiana* and *Cordyceps militaris*, both of which belong to the same lineage despite distinct (asexual alone versus sexual/asexual) lifecycles. In *B. bassiana*, SF1 consists of six members (Pr1 G, two Pr1A, and three Pr1B paralogues) but contains no homologue of Pr1I or Pr1 K while SF2 consists of four Pr1 F paralogues but lacks Pr1D, Pr1E, and Pr1 J homologues that coexist with unique Pr1 F in the two *Metarhizium* species. The nucleotide sequences of four *pr1 F* genes in *B. bassiana* are free of any intron (Supplementary Table S1) like the unique *pr1 F* in *M. anisopliae* complex [23,39], supporting the recognition of four Pr1F paralogues. Notably, Pr1C and Pr1G exist as orthologues in the two distinct fungal lineages, implicating a likelihood that both of them serve as conserved marks in the evolution of fungal insect pathogenicity. The *C. militaris* genome contains only one unnamed SF2 member (ATY62244) aside from homologues of Pr1C and five SF1 members in *B. bassiana*, suggesting that the complicated sexual/asexual cycles of *C. militaris* need no more SF2 proteases perhaps due to its specific infection to pupae. Those phylogenetically distinct SF1 and SF2 members in the two fungal lineages could have functionally differentiated.

**Figure 1.**
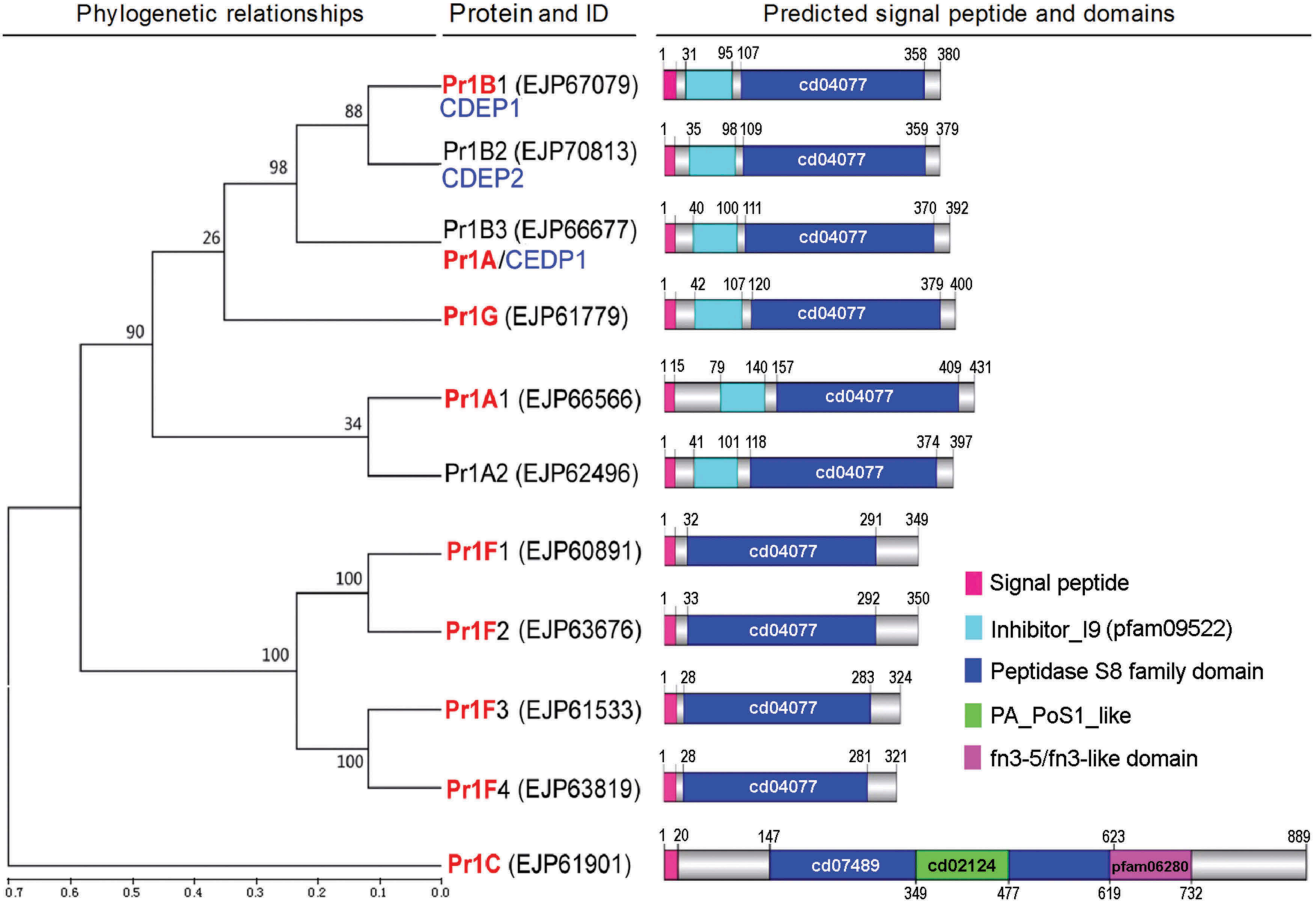
Phylogenetic relationships, sequence properties and revised nomenclature of 11 Pr1 proteases in *B. bassiana* genome [27]. The names of all Pr1 family members annotated (shown in red) and not annotated (shown in black) in the genome or disclosed in previous reports (shown in blue) are revised in terms of their phylogenetic relationships determined by bootstrap values of 1000 replications at nodes and genetic distances scaled with the neighbor-joining method in MEGA7 at http://www.megasoftware.net/. The N-terminal signal peptide and main domain(s) of each Pr1 family member are predicted online at http://www.cbs.dtu.dk/services/SignalP-3.0/and http://www.ncbi.nlm.nih.gov/Structure/, respectively.

The nomenclature of all Pr1 proteases annotated in the *B. bassiana* genome is revised with reference to phylogenetic ties and molecular properties including sequence identity (Supplementary Table S1). Illustrated in Figure 1, Pr1C and Pr1G follow genomic annotation, and four SF2 members are renamed Pr1F1–4. The Pr1 protease early reported [11] or studied as CDEP1 [16,18] in *B. bassiana* resembles the annotated Pr1B and is renamed Pr1B1. An unnamed Pr1 (379 aa) is phylogenetically closer to Pr1B1, identical to previously reported CDEP2 in molecular size [19], and renamed Pr1B2. One of two proteases annotated as Pr1A in the fungal genome is also reported as CDEP1 [20] but closer to Pr1B1/2 below the subclade level and hence renamed Pr1B3. Another annotated Pr1A and an unnamed Pr1 are closer to each other and renamed Pr1A1 and Pr1A2, respectively.

Also shown in Figure 1, all *B. bassiana* Pr1 proteases share an N-terminal signal peptide consisting of the first 15–20 amino acids. Ten class II (SF1/2) members are characterized by a large peptidase S8 family domain (cd04077), which contains an Asp/His/Ser catalytic triad typical for the proteinase K-like subtilisin family. Six SF1 members share another characteristic domain called peptidase Inhibitor I9 (pfam09522). The unique class I member Pr1C features a large domain similar to cd04077, a protease-associated (PA) domain (PA_PoS1_like) and an fn3_5 (fn3-like) domain, which exists in streptococcal C5a peptidase (SCP), a highly specific protease and adhesin/invasin [40–42]. The 11 Pr1 proteases are all predicted as extracellular enzymes with high probabilities (Supplementary Fig. S2) obviously due to an N-terminal signal peptide they share.

### Pr1 proteases essential and nonessential for extracellular Pr1 activity (EPA)

**Figure 2.**
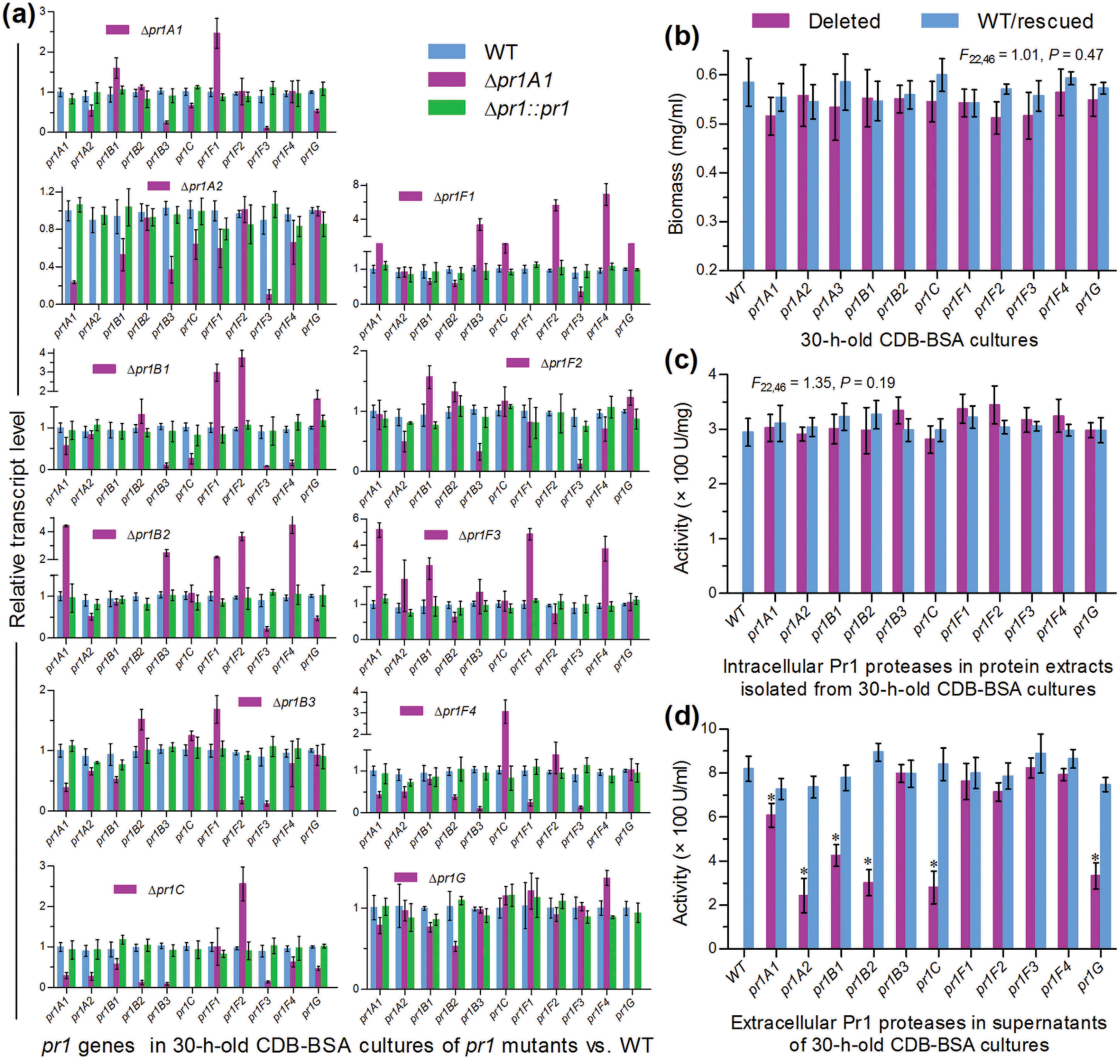
Impacts of each *pr1* deletion on transcriptional expression of 10 other *pr1* genes and total levels of intra- and extracellular Pr1 activities in *B. bassiana*. (a) Relative transcript levels of all *pr1* genes in the cDNA samples of given *pr1* mutants with respect to the WT standard. All cDNA samples were derived from the submerged cultures of a 10^7^ conidia/ml suspension in CDB-BSA incubated for 30 h at 25°C. Note that each deleted *pr1* gene was transcriptionally undetectable in the corresponding Δ*pr1* mutant. (b–d) Biomass levels, intracellular Pr1 activities, and extracellular Pr1 activities (EPA) quantified from the 30-h-old CDB-BSA cultures of all Δ*pr1* mutants and control strains (WT and complemented strains), the protein extracts of their cultures and the supernatants of their cultures, respectively. Note no significant variability among measurements of all tested strains in (b) and (c). The asterisked Δ*pr1* means in (d) differ significantly from those of the corresponding control strains unmarked (Tukey’s HSD, *P* < 0.05). Error bars: SD from three replicates.

Each of 11 *pr1* genes was deleted from the wild-type strain *B. bassiana* ARSEF 2860 (designated WT herein) by homogeneous recombination of its 5′ and 3′ coding/flanking fragments separated by *bar* marker and rescued in an identified deletion mutant by ectopic integration of a cassette consisting of its full-length sequence and *sur* marker, as described in Materials and methods. Expected recombinant events in all mutant strains were verified through PCR analysis (Supplementary Fig. S3). Real-time quantitative PCR (qPCR) analysis revealed an undetectable transcript level of each deleted *pr1* gene in the cDNA samples of the corresponding Δ*pr1* mutant and a transcriptional restoration of the same gene to the WT level by targeted gene rescue (Figure 2(a)), indicating a success in either the deletion or the complementation of each target gene.

Hyphal penetration through insect cuticle usually occurs from 24 to 36 h after conidial adhesion to insect integument [43]. For this reason, we assessed transcript levels of 10 *pr1* genes and total Pr1 activity in the submerged cultures of each Δ*pr1* mutant and its control (WT and complemented) strains generated from 30-h incubation of a 10^7^ conidia/ml suspension in Czapek-Dox broth (CDB) containing 0.3% bovine serum albumin (BSA) as sole nitrogen source and enzyme inducer. Also shown in Figure 2(a), each *pr1* deletion resulted in marked down- or up-regulation of some other *pr1* genes. Those differentially expressed genes in the absence of a given *pr1* implicated transcriptional interactions among the Pr1 family members. Biomass levels (Figure 2(b)) measured from the 30-h-old CDB-BSA cultures showed an insignificant variability (*F*_22,46_ = 1.01, *P* = 0.47 in one-way analysis of variance) among all tested strains. Intracellular Pr1 activities (Figure 2(c)) quantified from the protein extracts of the submerged cultures also exhibited no significant variability (*F*_22,46_ = 1.35, *P* = 0.19), suggesting a total level of intracellular Pr1 accumulation independent of each *pr1* deletion. By contrast, total EPA levels quantified from the supernatants of such cultures were averagely lowered by 70% in Δ*pr1A2*, 66% in Δ*pr1C*, 63% in Δ*pr1B2*, 60% in Δ*pr1G*, 48% in Δ*pr1B1*, and 25% in Δ*pr1A1* but unaffected (Tukey’s HSD, *P* > 0.05) in Δ*pr1B3* and four Δ*pr1 F* mutants in comparison to the WT strain (Figure 2(d)). Notably, reduced EPA often concurred with decreased transcripts of *pr1 C, pr1 G* and/or some other SF1 genes in the mentioned Δ*pr1* mutants. For instance, all SF1 genes were differentially suppressed in the Δ*pr1 C* mutant severely compromised in total EPA. At least two SF1 genes and *pr1C* were down-regulated in the rest EPA-reduced Δ*pr1* mutants. In the Δ*pr1B3* and four Δ*pr1F* mutants, unaffected EPA levels were often concurrent with negligible transcript changes of *pr1C* and critical SF1 genes or a counterbalance of their differential expressions, but seemingly not associated with down- or up-regulation levels of some other SF2 genes. All of the mentioned transcript and EPA changes were restored in the complemented strains. Thus, total EPA reduction was attributed to not only the absence of each critical *pr1* but also the suppressed expression of some other critical genes including *pr1C, pr1A2, pr1B1, pr1B2* and/or *pr1G*.

**Figure 3.**
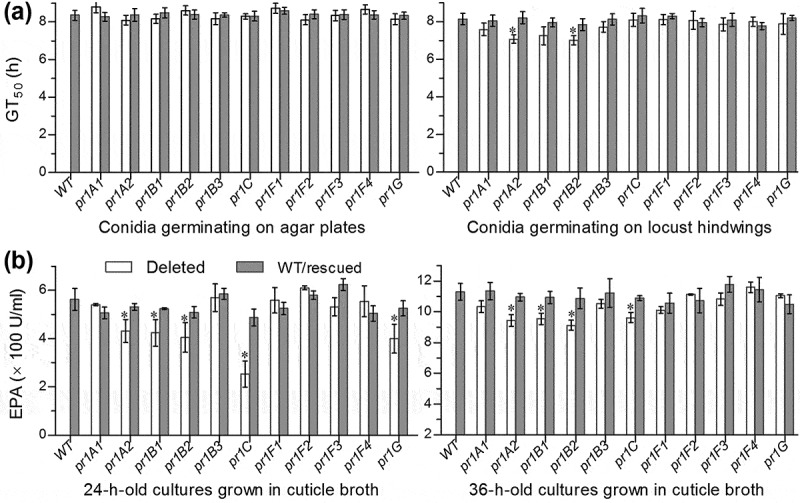
Impacts of each *pr1* deletion on germination of conidia and secretion of Pr1 proteases in the presence of insect cuticle. (a) GT_50_ (h) estimates of all tested strains for 50% conidial germination on agar plates and locust hindwings at optimal 25°C. (b) Total levels of extracellular Pr1 activities (EPA) quantified from the supernatants of the submerged cultures incubated 24 and 36 h at 25°C in 0.625% M-100 salt solution containing 1% ground locust cuticle as a sole nutrient source. Each culture was generated from the shaking incubation of a 10^6^ conidia/ml suspension in the cuticle broth. The asterisked Δ*pr1* means in each bar chart differs significantly from those of the corresponding control strains unmarked (Tukey’s HSD, *P* < 0.05). Error bars: SD from three replicates.

Further, we compared conidial germination rates of all Δ*pr1* mutants and control strains on agar plates and locust (*Locusta migratoria manilensis*) hind wings and assessed their EPA levels in the supernatants of the submerged cultures grown in locust cuticle broth (CB) containing 1% ground cuticle as a sole nutrient source in 0.625% M-100 salt solution [44]. As a result, the estimates of median germination time (GT_50_) for 50% germination at 25°C had little variability among all strains tested on the plates (*F*_22,46_ = 1.73, *P* = 0.058; Figure 3(a)). Conidial germination rates indicated by GT_50_s on locust hind wings were also similar among nine Δ*pr1* mutants and their control strains (Tukey’s HSD, *P* > 0.05) but slightly accelerated in the absence of *pr1A2* or *pr1B2* (Figure 3(b)). Total EPA levels assessed from the supernatants of the 24-h-old cultures initiated with 10^6^ conidia/ml CB on average decreased by 55% in Δ*pr1C*, 29% in Δ*pr1G*, 28% in Δ*pr1B2*, 25% in Δ*pr1B1* and 23% in Δ*pr1A2*, but were insignificantly affected in other Δ*pr1* mutants in comparison to the control strains (Figure 3(c)). The decreased magnitudes diminished to only 15–19% in the 36-h-old CB cultures of the mentioned mutants except Δ*pr1G* showing an insignificant change (Figure 3(d)). However, the variability of such assessments among all tested strains became marginal (*F*_22,46_ = 1.83, *P* = 0.05) by the end of 48-h cultivation and insignificant (*P* > 0.05 for *F* test) from then on (data not shown).

Taken together, class I Pr1C and four class II SF1 members (Pr1A2, Pr1B1, Pr1B2, and Pr1G) contributed significantly to total EPA, contrasting to little contribution from each of five other class II members (SF1 Pr1B3 and SF2 Pr1F1–4) in *B. bassiana*. The contribution of Pr1A1 to total EPA was smallest in the CDB-BSA culture and insignificant in the CB culture, hinting at a similarity of this SF1 member to functionally redundant Pr1B3 and four Pr1F paralogues.

### EPA-required Pr1 proteases are essential for fungal pathogenicity but nonessential for virulence-related cellular events post-infection

**Figure 4.**
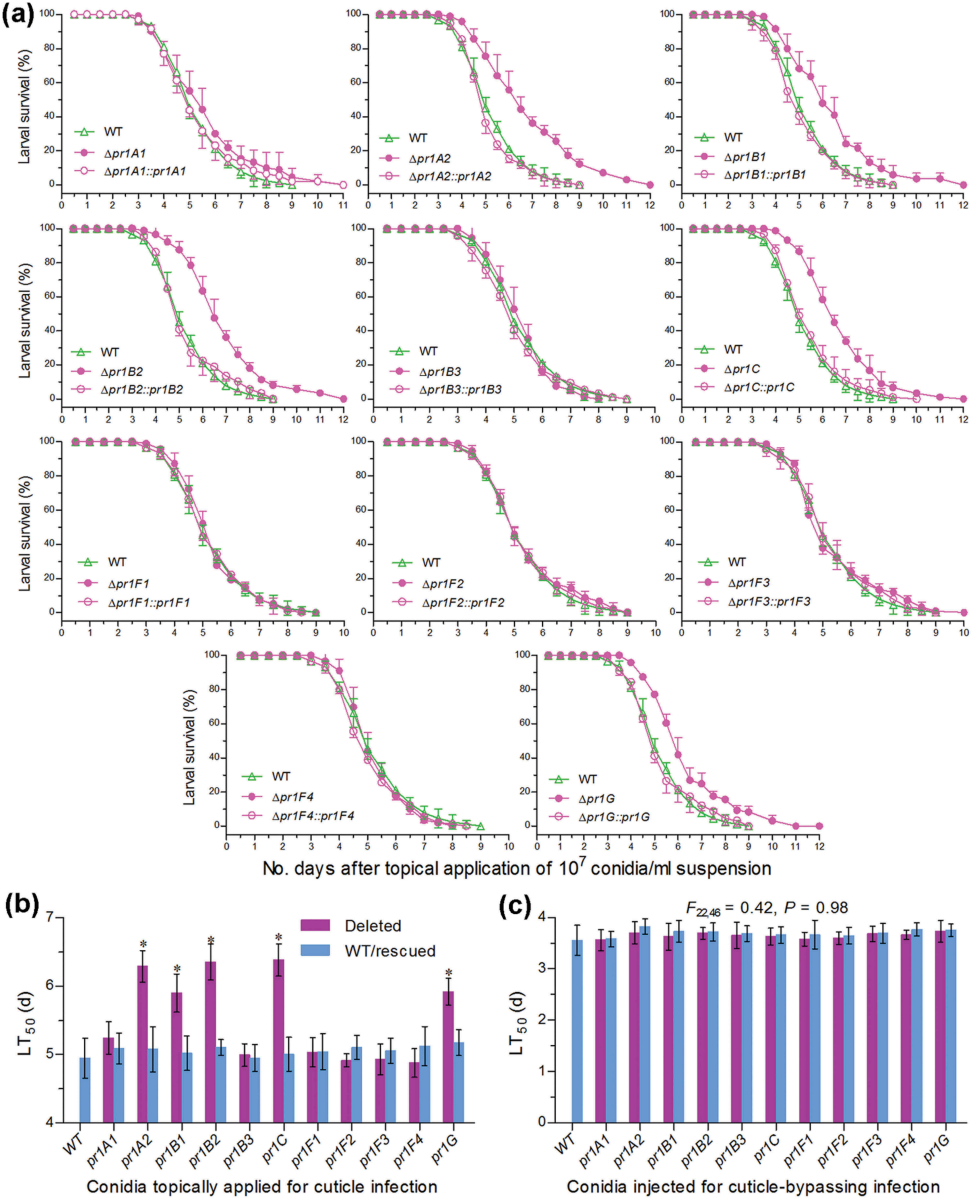
Impacts of each *pr1* deletion on the virulence of *B. bassiana*. (a, b) Survival trends of *G. mellonella* larvae after topical application (immersion) of a 10^7^ conidia/ml suspension for normal cuticle infection and LT_50_ estimates as virulence indices of all tested strains estimated from time-morality trends. The asterisked Δ*pr1* LT_50_ s differ significantly from those of the corresponding control strains unmarked (Tukey’s HSD, *P* < 0.05). (c) LT_50_ estimates of all tested strains against *G. mellonella* larvae inoculated by intrahaemocoel injection of ~500 conidia per larva for cuticle¯bypassing infection (see Figure S4 for survival trends of injected larvae). Note no significant variability among the LT_50_ s of all tested strains. Error bars: SD from three replicates.

Two types of bioassays were performed to distinguish roles of 11 Pr1 proteases in sustaining insect pathogenicity and virulence-related cellular events after the fifth-instar larvae (~300 mg in body weight *per capita*) of the model insect *Galleria mellonella* were inoculated by topical application (immersion) of a 10^7^ conidia/ml suspension for normal cuticle infection or intrahaemocoel injection of ~500 conidia per larva for cuticle-bypassing infection. The larvae infected by the deletion mutants of five EPA-required *pr1* genes survived longer than those infected by their control strains via cuticular penetration but such differences disappeared in other six Δ*pr1* mutants via the cuticle infection (Figure 4(a)) and in all Δ*pr1* mutants and control strains tested via the cuticle-bypassing infection (Supplementary Fig. S4). Consequently, estimates of medial lethal time (LT_50_) through the cuticle infection were significantly prolonged by 29% in Δ*pr1C*, 28% in Δ*pr1B2*, 27% in Δ*pr1A2*, 20% in Δ*pr1G*, and 19% in Δ*pr1B1* but did not differ significantly between control strains and the rest Δ*pr1* mutants (Figure 4(b)), including Δ*pr1A1* whose EPA was moderately lowered in the CDB-BSA culture but unaffected in the CB culture. The LT_50_ estimates through the cuticle-bypassing infection were identical for all deletion mutants and control strains (*F*_22,46_ = 0.42, *P* = 0.98) against the insect species (Figure 4(c)). Demonstrated with these data, only *pr1C* and four SF1 genes functioned in a hyphal invasion into host via cuticular penetration and were individually responsible for a 19–29% change in virulence of *B. bassiana*. However, none of the studied *pr1* genes was involved in the virulence-related events after entry into host hemocoel. This inference is supported by further observations and experimental data as follows.

**Figure 5.**
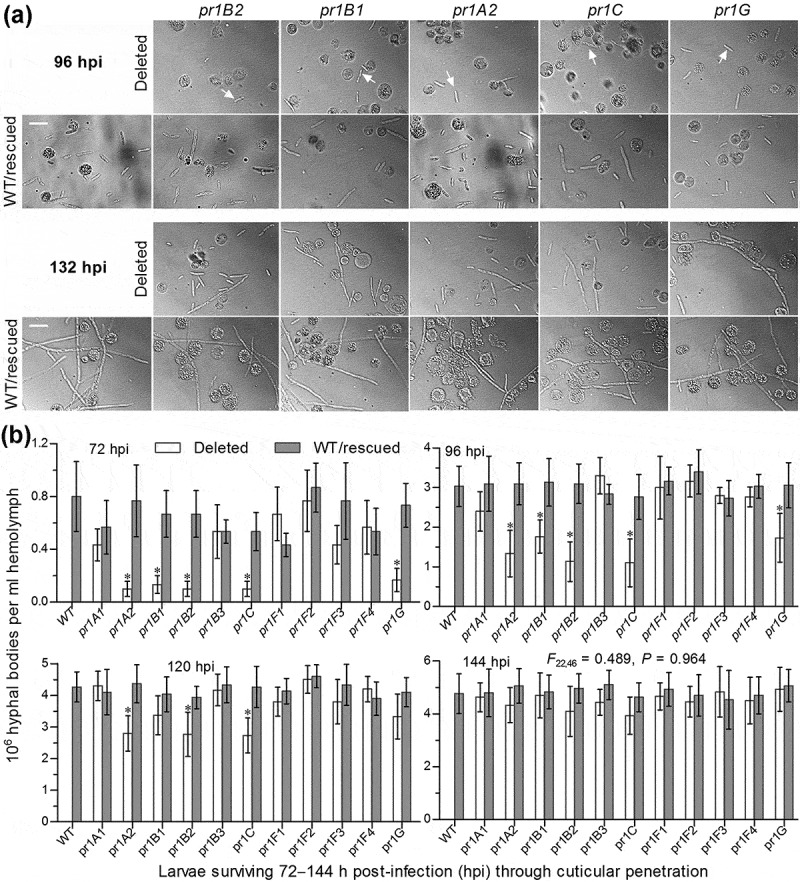
Impacts of each *pr1* deletion on development of hyphal bodies in the hemolymph of *G. mellonella* larvae after topical application (immersion) of a 10^7^ conidia/ml suspension for normal cuticle infection. (a) Microscopic images (scale = 20 μm) for abundance of hyphal bodies (arrowed) in the hemolymph samples taken from the larvae surviving 96 and 132 h post-infection (hpi). Spherical and subspherical cells are insect hemocytes. (b) Concentrations of hyphal bodies quantified from the hemolymph samples taken from the larvae surviving 72–144 hpi. The asterisked Δ*pr1* means differ significantly from those of the corresponding control strains unmarked (Tukey’s HSD, *P* < 0.05). Error bars: SD from three replicates.

First, we examined hemolymph samples taken from surviving larvae after the cuticle infection and found that, compared to the control strains, the five mentioned Δ*pr1* mutants suffered a marked delay in the development of hyphal bodies in host hemocoel (Figure 5(a)). The concentrations of hyphal bodies in the samples from the larvae infected by these mutants decreased by 79–88% and 42–63% at 72 and 96 h post-infection (hpi) respectively (Figure 5(b)). Such significant reductions diminished to ~35% only in Δ*pr1A2*, Δ*pr1B2* and Δ*pr1C* at 120 hpi and disappeared in all Δ*pr1* mutants at 144 hpi (*F*_22,46_ = 0.49, *P* = 0.96). In the five Δ*pr1* mutants, obviously, the retarded development of hyphal bodies critical for the acceleration of host mummification by yeast-like budding was associated with delayed entries into host hemocoel due to reduced EPA levels necessary for cuticle degradation.

Second, we compared growth rates and conidiation levels of all fungal strains since key activators of the central developmental pathway are indispensable for hyphal development and insect pathogenicity of *B. bassiana* [33]. As a result, all Δ*pr1* mutants behaved as well in radial growth as did their controls strains at the optimal regime of 25°C in a light/dark cycle of 12:12 h on the plates of rich SDAY (Sabouraud dextrose agar plus yeast extract), 1/4 SDAY (one-fourth of SDAY nutrients), minimal CDA (i.e., CDB plus agar) and several CDAs amended with different carbon or nitrogen sources or in the absence of carbon source, nitrogen source or both (Supplemental Figure S5(a)). Conidial yields and biomass levels quantified, respectively, from the 8- and 7-day-old SDAY cultures initiated by spreading 100 μl of a 10^7^ conidia/ml suspension per plate (9 cm diameter) at the optimal regime showed no significant difference between each Δ*pr1* mutant and its control strains (Tukey’s HSD, *P* > 0.05; Supplemental Figure S5(b)). Apparently, each Pr1 protease played no role in either radial growth on scant substrata, such as oligotrophic insect integument, or hyphal development and aerial conidiation on SDAY, a standard medium for conventional cultivation of fungal insect pathogens.

Third, fungal responses to various stress cues encountered during infection and after entry into host hemocoel are critical for successful infection and host mummification [30]. Encountered stress cues include high osmolarity in trehalose-concentrated host hemolymph, oxidative stress of reactive oxygen species generated from host immunity defense, and behavioral fever (elevated temperature) of early infected host [30,45,46]. For this reason, we assayed cellular responses to two osmotic agents (NaCl and sorbitol), two oxidants (menadione and H_2_O_2_) and three cell wall stressors (Congo red, calcofluor white, and SDS) during colony growth on CDA. As a result, all Δ*pr1* mutants and control strains were equally responsive to each of the tested stress cues due to their similarities in colony size and morphology after a 7-day co-incubation with each chemical at 25°C (Supplemental Figure S6). Moreover, exposing 2.5-day-old SDAY colonies to a 42°C heat shock for 3, 6 and 9 h also resulted in similarities of their colonies after 4.5-day growth recovery at 25°C. All colony measurements (data not shown) revealed null responses of all Δ*pr1* mutants to hyperosmotic, oxidative, cell wall perturbing and thermal stresses. Thus, none of the Pr1 proteases played a role in the fungal responses to the stress cues tested *in vitro*, hinting at no substantial role of each in the fungal resistance to possible stress cues encountered during host infection or post-infection.

**Figure 6.**
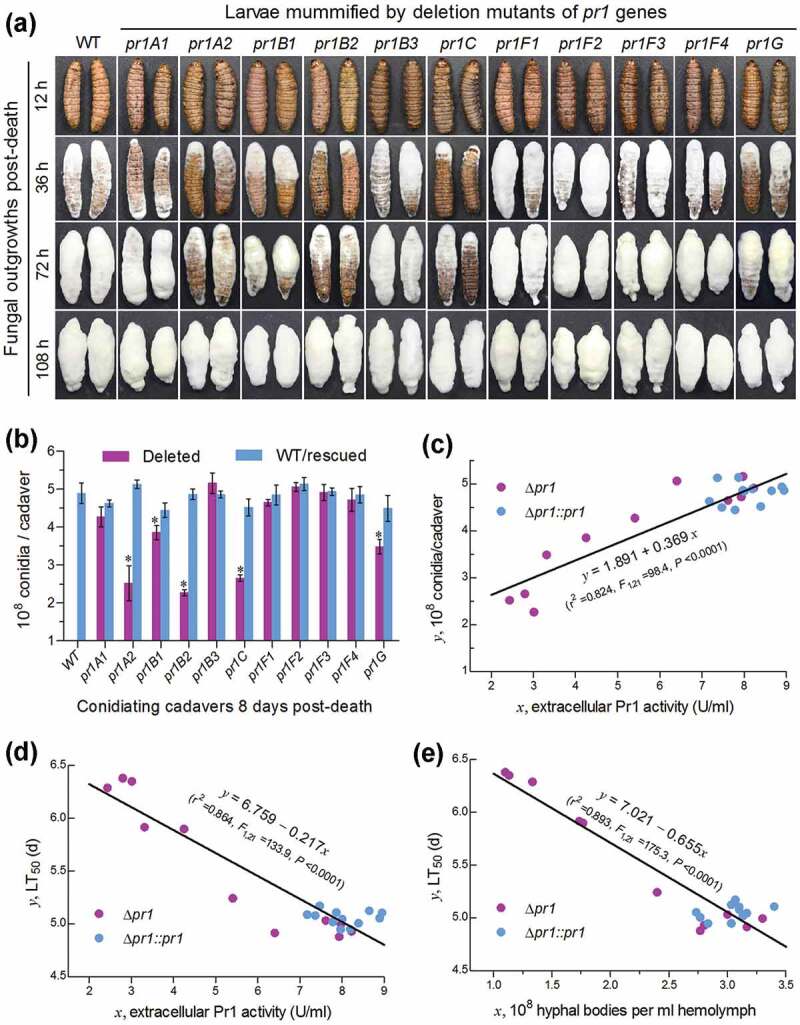
Impacts of each *pr1* deletion on the hyphal growth and conidiation of *B. bassiana* in mummified insects. (a) Images of fungal outgrowth rates on *G. mellonella* cadavers mummified by Δ*pr1* mutants and their control strains. (b) Conidial yields measured from the surfaces of cadavers 8 days post-death. The asterisked Δ*pr1* means differ significantly from those of the corresponding control strains unmarked (Tukey’s HSD, *P* < 0.05). Error bars: SD from three replicates (three cadavers per replicate). (c–d) Linear correlations of paired phenotypic parameters indicating a significant dependence of either cadaver conidiation level or cuticle infection-caused LT_50_ on extracellular Pr1 activity and of the LT_50_ on the concentration (development) of intrahaemocoel hyphal bodies among all Δ*pr1* mutants and control strains.

Fourth, we examined fungal outgrowths on insect cadavers and quantified their conidial yields, which are crucial for fungal survival, dispersal, and infection cycle in host habitats. The five Δ*pr1* mutants attenuated in virulence grew out of insect cadavers slower than the WT strain and the rest virulence-unaffected Δ*pr1* mutants during a period of the first 3 days post-death (Figure 6(a)), indicating a delay in their penetrating again through insect cuticle for outgrowth. Moreover, conidial yields of the five mutants quantified from insect cadavers 8 days post-death decreased significantly by 21–54% in comparison to the corresponding yields of their control strains (Figure 6(b)), implicating a link of each essential Pr1 protease to fungal survival, dispersal, and infection cycle in host habitats.

Finally, and interestingly, linear regression analysis of paired phenotypes from all tested strains revealed significant correlations between total EPA level and conidial yield on insect cadavers (r^2^ = 0.82; Figure 6(c)) or LT_50_ through the cuticle infection (r^2^ = 0.86; Figure 6(d)) and between LT_50_ and concentration of hyphal bodies in the hemolymph samples taken 4 days post-infection (r^2^ = 0.89; Figure 6(e)) or 3 days post-infection (r^2^ = 0.69, *P* < 0.0001; data not shown).

## Discussion

Taken our experimental results into account, class I Pr1C in and class II-SF1 Pr1A2, Pr1B1, Pr1B2, and Pr1G are essential for the maintenance of total EPA required for cuticle degradation during hyphal invasion of *B. bassiana* into host insect and serve as conserved molecules that mark the fungal pathogenicity to the broadest host spectrum. By contrast, Pr1A1 and Pr1B3 in SF1 and four Pr1F paralogues in SF2 have no substantial role in the fungal infection to insect through cuticular penetration due to a negligible or insignificant contribution to total EPA, and hence are possible evolution remnants of insect pathogenicity in *B. bassiana*. Aside from the unique role in the cuticle degradation, the five conserved Pr1 proteases recognized in this study play little role in post-infection cellular events crucial for the fungal virulence, such as hyphal growth, dimorphic transition, and cellular responses to stress cues encountered during host infection or post-infection. This is supported by no differences of the examined phenotypes between 11 Δ*pr1* mutants and control strains, including median lethal action via intrahaemocoel injection. These findings provide the first referable base for insight into molecular evolution of insect pathogenicity marked by the conserved Pr1 proteases in *B. bassiana*, an ancient insect-pathogenic fungus relative to *M. anisopliae* complex [2].

Among the five conserved Pr1 proteases, Pr1C and Pr1G exist as orthologues in the two fungal lineages and proved to be crucial for both total EPA (particularly in the cuticle cultures) and insect pathogenicity of *B. bassiana*. This implicates that Pr1C and Pr1G could also function in determining insect pathogenicity of other fungal lineages, including *M. anisopliae* complex undergoing speciation [24] and molecular evolution toward diversification of Pr1 family members [37]. Pr1A2, Pr1B1, and Pr1B2 are functionally similar to Pr1C and Pr1G in the maintenance of total EPA and insect pathogenicity. The significantly linear correlations revealed in this study reinforce tight links of hyphal invasion, lethal action and infection cycle to total EPA levels depending on the five essential Pr1 proteases in *B. bassiana*. The roles of these functional Pr1 proteases in *B. bassiana* are similar to those of Pr1A and Pr1B in *M. anisopliae*, in which both Pr1 activity and insect pathogenicity were reduced by knockout mutation of unique *pr1A* or *pr1B* [14]. Since most Pr1 proteases have not been characterized yet in *M. anisopliae* complex, it is hard to expand a discussion on functional similarities and differences of all Pr1 family members between the two fungal lineages serving as main sources of fungal insecticides [1]. We speculate that, aside from Pr1A and Pr1B, some of those class II proteases (Pr1D, Pr1E, Pr1I, Pr1J, and Pr1K) not existing in *B. bassiana* could function in the young insect pathogens evolving in or evolved from *M. anisopliae* complex, such as *M. robertsii* and *M. acridum* [25]. In this study, no perceivable phenotype change in the deletion mutants of *pr1A1, pr1B3,* and four *pr1F* genes indicates that their individual roles are insignificant in *B. bassiana* but does not necessarily imply that their collective roles are also insignificant. Fungal virulence may vary with host insect species [38]. The significant and insignificant contributions of the examined *pr1* genes to virulence against *G. mellonella* in this study might change in some other insects that enable to generate immunity-induced antifungal molecules counteracting specific Pr1 proteases in response to fungal infection. Only are those proteases considered as virulence factors that are either not or partially inhibited by the corresponding protease inhibitors and antifungal peptides in infected host [3,5,36]. The six Pr1 proteases not functional in cuticle degradation in this study could have encountered corresponding host-derived protease inhibitors as previously shown to be produced in the hemolymph of infected *G. mellonella* [35]. Nonetheless, it is worthwhile to clarify whether the unique Pr1F and three other SF2 proteases function individually or collectively in *M. anisopliae* complex. Comparisons of all functional Pr1 family members between *B. bassiana* and different lineages of fungal insect pathogens including *M. anisopliae* complex may help to gain in-depth insight into the molecular evolution of fungal insect pathogenicity and to promote utilization of potent *pr1* genes for the acceleration of fungal invasion into host. Aside from the Pr1 family, functionally similar thermolysin-like metalloproteases and other types of extracellular enzymes involved in cuticle degradation, such as chitinases and lipases, are also important for the success of fungal cuticle infection [3–5]. Whether these enzymes contribute individually or collectively to fungal virulence against insects warrants more studies.

Fungal virulence is an overall output of many extra- and intrahaemocoel cellular events regulated by multiple cellular signaling pathways [30,31,33,34]. With no doubt, functional Pr1 proteases aid fungal invasion by cuticle degradation and collectively are essential for fungal insect pathogenicity. However, the five functional Pr1 proteases were not involved in any other virulence-related cellular events and hence individually contributed only 19–29% to the fungal virulence via normal cuticle infection based on an LT_50_ prolonged in the absence of each. Such a contribution is consistent with previous reports. For instance, overexpression of *pr1A* or *CDEP1* (*pr1B1*) in engineered *M. anisopliae* or *B. bassiana* strains shortened the LT_50_ by 25% or 12.5% against *Manduca sexta* [13] or *G. mellonella* [16]. The present and previous studies demonstrate that even the functional Pr1 proteases individually contribute a limited part to the fungal virulence. This is in contrast with a complete loss of fungal ability to infect the host through cuticular penetration in the absence of a single gene, such as those encoding the DnaJ domain-containing protein Mas5 [47], the Na^+^/H^+^ antiporter Nhx1 [48], the Fus3-cascaded components of mitogen-activated protein kinase (MAPK) pathway [49,50], the histone acetyltransferase Gcn5 [51] and the developmental activator BrlA or AbaA [33] in *B. bassiana*. These characterized genes are involved in not only hyphal growth and invasion into host but also post-infection cellular events to accelerate host mummification to death. From this point of view, it is reasonable to consider functional Pr1 proteases as a fungal invasion- or pathogenicity-augmenting factors rather than virulence factors due to their roles strictly limited to insect cuticle degradation.

## Materials and methods

### Bioinformatic analysis of Pr1 proteases in B. bassiana

The sequences of Pr1A–K early named in *M. anisopliae* complex [15,23] were used as queries to search through the genomes of *M. robertsii* [24], *C. militaris* [26] and *B. bassiana* [27] by BLASTp analysis at http://blast.ncbi.nlm.nih.gov/Blast.cgi/. All Pr1 homologues found in the *Beauveria/Cordyceps* and *Metarhizium* lineages were clustered by phylogenetic analysis with a neighbor-joining method in MEGA7 at http://www.megasoftware.net/. Each of 11 Pr1 homologues in *B. bassiana* was structurally analyzed at http://www.ncbi.nlm.nih. gov/Structure/, followed by predicting the presence/absence of an N-terminal signal peptide from each of their amino acid sequences at http://www.cbs.dtu.dk/services/SignalP-3.0/ and the probabilities for intracellular, extracellular and transmembrane activities of each Pr1 at http://www.cbs.dtu.dk/services/TMHMM/. Their molecular properties were analyzed with the online ProtParam program (http://expasy.org/tools/protparam.html).

### Generation of paired pr1 mutants

Each *pr1* gene was deleted from the WT strain by homogenous recombination of its 5′ and 3′ fragments separated by *bar* maker (p0380-5′*x*-bar-3′*x*, where *x* denotes one of 11 *pr1* genes (Supplementary Table S2) and complemented into an identified deletion mutant by ectopic integration of a cassette consisting of its full-length sequence and *sur* marker (p0380-sur-*x*) through *Agrobacterium*-mediated transformation following our previous protocols [52]. Briefly, the deletion plasmid was constructed by amplifying the 5′ and 3′ coding/flanking fragments (~1500 bp *per capita*) of each *pr1* from the WT DNA with paired primers under the action of La*Taq* DNA polymerase from Promega (Madison, MI, USA) and inserting the fragments into appropriate enzyme sites of p0380-bar. The complementary plasmid was constructed by amplifying a full-length coding/flanking sequence of each *pr1* from the WT DNA and inserting it into p0380-sur-gateway to exchange for the gateway fragment. After transformation, putative mutant colonies were screened based on the *bar* resistance to phosphinothricin (200 μg/ml) or the *sur* resistance to chlorimuron ethyl (10 μg/ml), followed by PCR and qPCR analyses with paired primers (Supplementary Table S2) to verify the expected recombination events. Eleven pairs of positive Δ*x* and Δ*x::x* mutants were selected after five rounds of cultivation on SDAY (4% glucose, 1% peptone 1.5% agar plus 1% yeast extract) at the optimal regime and evaluated in parallel with the WT strain in the following experiments of three independent cultures (replicates) or samples from the cultures.

### Transcriptional profiling

For all Δ*pr1* mutants and their control strains, 50 ml aliquots of a 10^7^ conidia/ml suspension in CDB-BSA [3% sucrose, 0.3% BSA (substitute of 0.3% NaNO_3_ as sole nitrogen source in standard CDB), 0.1% K_2_HPO_4_, 0.05% KCl, 0.05% MgSO,_4_ and 0.001% FeSO_4_] were incubated 30 h on a shaking bed (150 rpm) at optimal 25°C. Total RNAs were extracted from the resultant cultures with RNAiso Plus Kit (TaKaRa, Dalian, China) and reversely transcribed into cDNAs with PrimeScriptH^RT^ reagent Kit (TaKaRa). Three samples of each cDNA were used as templates to assess transcript levels of 11 *pr1* genes in each strain through qPCR with paired primers (Supplementary Table S2) under the action of SYBR® Premix Ex Taq^TM^ (TaKaRa). The fungal β-actin gene was used as an internal control. A threshold-cycle (2^–ΔΔCT^) method was used to compute relative transcript levels of all *pr1* genes in each Δ*pr1* or Δ*pr1::pr1* mutant with respect to the WT standard. Each qPCR was repeated three times using cDNA samples derived from independent cultures.

### Assays for total Pr1 activities

Total levels of EPA and intracellular Pr1 activity in the 30-h-old CDB-BSA cultures of each strain as aforementioned were quantified following previous protocols [47,53]. Briefly, each of the cultures was separated into supernatant and hyphal cells by centrifugation at 4°C, and the hyphal biomass was assessed after 3-h drying at 70°C. Total EPA in each supernatant (crude extract) was quantified from a system of 50 µl 1 mM substrate [succinyl-(alanine)_2_-proline- phenylalanine-*p*-nitroanilide; Sigma], 850 µl 15 mM Tris-HCl buffer (pH 8.5) and 100 µl each extract or heat-inactivated extract (control). The reaction lasted 1 h at 28°C and terminated by adding 250 µl of 30% acetic acid. After standing 15 min in ice, the product was centrifuged at 1250 *g* for 5 min at 4°C, followed by reading optical density at 410 nm (OD_410_). To quantify the total activity of intracellular Pr1 proteases, fresh hyphal cells from each culture were ground in liquid nitrogen, suspended in 50 mM phosphate buffer (pH 7.4) and centrifuged by 12 000 × g at 4°C. The protein concentration in the extract was determined with BCA Protein Assay Kit (KeyGen, Nanjing, China), followed by reading the OD_410_ value from the reaction system as described above. One unit of enzyme activity was defined as an enzyme amount required for an OD_410_ increase by 0.01 after 1 h reaction of each extract versus control. Total levels of EPA and intracellular Pr1 activity were expressed as U/ml supernatant and U/mg protein extract, respectively.

For insight into the secretion of Pr1 proteases by hyphal cells on insect cuticle, 50 ml aliquots of a 10^6^ conidia/ml suspension in CB (0.625% M-100 salt solution containing 1% ground locust cuticle as sole nutrient source) [44] were incubated 120 h on the shaking bed at optimal 25°C. During the period of incubation, the same reaction system as described above was used to quantify total EPA levels from the supernatants of three CB cultures per strain at 12-h interval from 24 h onwards.

### Phenotypic experiments

For all strains, 1 μl aliquots of a 10^6^ conidia/ml suspension were centrally spotted for initiation of colony growth on the plates of SDAY, 1/4 SDAY (amended with 1/4 of each SDAY nutrient), CDA (SDB plus 1.5% agar) and CDAs amended by deleting 3% sucrose, 0.3% NaNO_3_ or both from the standard CDA, replacing the carbon source with 3% of trehalose, maltose, fructose, lactose, mannitol, glycerol or acetate (NaAc), and replacing the nitrogen source with 0.3% of NH_4_Cl, NaNO_2_ or NH_4_NO_3_, respectively. After a 7-day incubation at 25°C, fungal colonies on different media were photographed, and the mean diameter of each colony was estimated as a growth index of each strain on each medium with two measurements taken perpendicular to each other across the colony center.

The same spotting method was used to initiate fungal colonies on the plates of CDA alone or supplemented with one of the chemical stressors at a sensitive concentration: (i) NaCl (1 M) or sorbitol (1 M) for osmotic stress; (ii) menadione (0.04 mM) or H_2_O_2_ (2 mM) for oxidative stress; and (iii) Sodium dodecyl sulfate (SDS, 0.1 mg/ml), Congo red (6 μg/ml) or calcofluor white (5 μg/ml) for cell wall perturbing stress. After a 7-day incubation at 25°C, colonies grown under different stresses were photographed, followed by measuring their diameters as aforementioned. To assess cell response to heat stress, 2.5-day-old SDAY colonies initiated as above at 25°C were exposed to a heat shock of 42°C for 3, 6, and 9 h, respectively. The heat-shocked colonies were immediately transferred to 25°C for 4.5-day growth recovery, followed by photograph collection and diameter estimation.

For assessment of conidiation capacity, SDAY cultures were initiated by spreading 100 μl of a 10^7^ conidia/ml suspension per plate (9 cm diameter) and incubated for 8 days at the optimal regime of 25°C in a light/dark cycle of 12:12 h. Three plugs (5 mm diameter) were taken from each plate culture using a cork borer. All conidia of each plug were released into 1 ml of 0.02% Tween 80 by supersonic vibration. Conidial concentration in the resultant suspension was assessed with a hemocytometer and converted to the number of conidia per unit area (cm^2^) of plate culture. Additionally, each strain was grown on cellophane-overlaid SDAY plates for 7 days as mentioned above. Biomass level assessed from each of the plate cultures after drying was used as a reference to the conidial yield measured.

Conidial germination rate of each strain was assayed by spreading 80 μl aliquots of a 10^7^ conidia/ml suspension on agar plates (containing 2% sucrose and 0.5% peptone) or ~5 μl aliquots of the same suspension on locust hindwings, followed by incubation at 25°C until germination percentage had no more change. During the period of incubation, germinated, and non-germinated conidia on each plate or hindwing were counted from three fields of view under a microscope. GT_50_ (h) for 50% germination of each strain at 25°C was estimated as an index of conidial germination rate by modeling analysis of the resultant time-germination trend in each of three replicates.

Conidial virulence of each strain to the fifth instar larvae of *G. mellonella* were assayed with two inoculation methods. Briefly, three groups of ~35 larvae were separately immersed for 10 s in 30 ml aliquots of a 10^7^ conidia/ml suspension to initiate normal infection through cuticular penetration. Alternatively, 5 μl of a 10^5^ conidia/ml suspension was injected into the hemocoel of each larva in each group for cuticle-bypassing infection. All treated groups were maintained at 25°C and monitored every 12 or 24 h for survival/mortality records until all larvae died from mummification. LT_50_ was estimated through probit analysis of the time-mortality trend in each group. During the period of monitoring, hemolymph samples were taken from surviving larvae and examined for the presence/absence and abundance of hyphal bodies under a microscope. The concentration of hyphal bodies in each hemolymph sample was estimated with a hemocytometer. Cadavers mummified by each strain were maintained for 8 days at the optimal regime for fungal outgrowths, which were observed or photographed in a time-course manner. Conidia formed on the surfaces of three cadavers 8 days post-death were collectively released into 10 ml of 0.02% Tween 80 by supersonic vibration, followed by assessing the conidial concentration of the resultant suspension with a hemocytometer and converting it to the number of conidia produced per cadaver. The mean conidial yield of each strain on cadaver surfaces was estimated from three assessments (three cadavers *per capita*).

### Data analysis

All measurements and fitted parameters from the experiments of three independent replicates were subjected to one-way (strain) analysis of variance, followed by Tukey’s honestly significant difference (HSD) test for phenotypic differences between each Δ*pr1* mutant and its control strains. Paired phenotypes of all tested strains were analyzed to reveal a linear correlation of total EPA with the LT_50_ through normal cuticle infection or the conidiation level on insect cadavers and of the LT_50_ with the concentration of hyphal bodies in insect hemolymph at different sampling occasions.

## Supplementary material

The supplementary data for this article can be accessed here.
