# Supplementary material for: Subtilisin-like Pr1 proteases marking the evolution of pathogenicity in a wide-spectrum insect-pathogenic fungus
Source: Virulence. 2020 Apr 23;11(1):365–80. doi: 10.1080/21505594.2020.1749487 (PMC7199741; doi:10.1080/21505594.2020.1749487)
Supplement: Supplemental Material [file kvir-11-01-1749487-s001.docx]

**Supplementary Material**

**Subtilisin-like Pr1 proteases marking evolution of pathogenicity in a wide-spectrum insect-pathogenic fungus**

Ben-Jie Gao, Ya-Ni Mou, Sen-Miao Tong, Sheng-Hua Ying, and Ming-Guang Feng*

(*Corresponding author: E-mail: mgfeng@zju.edu.cn)


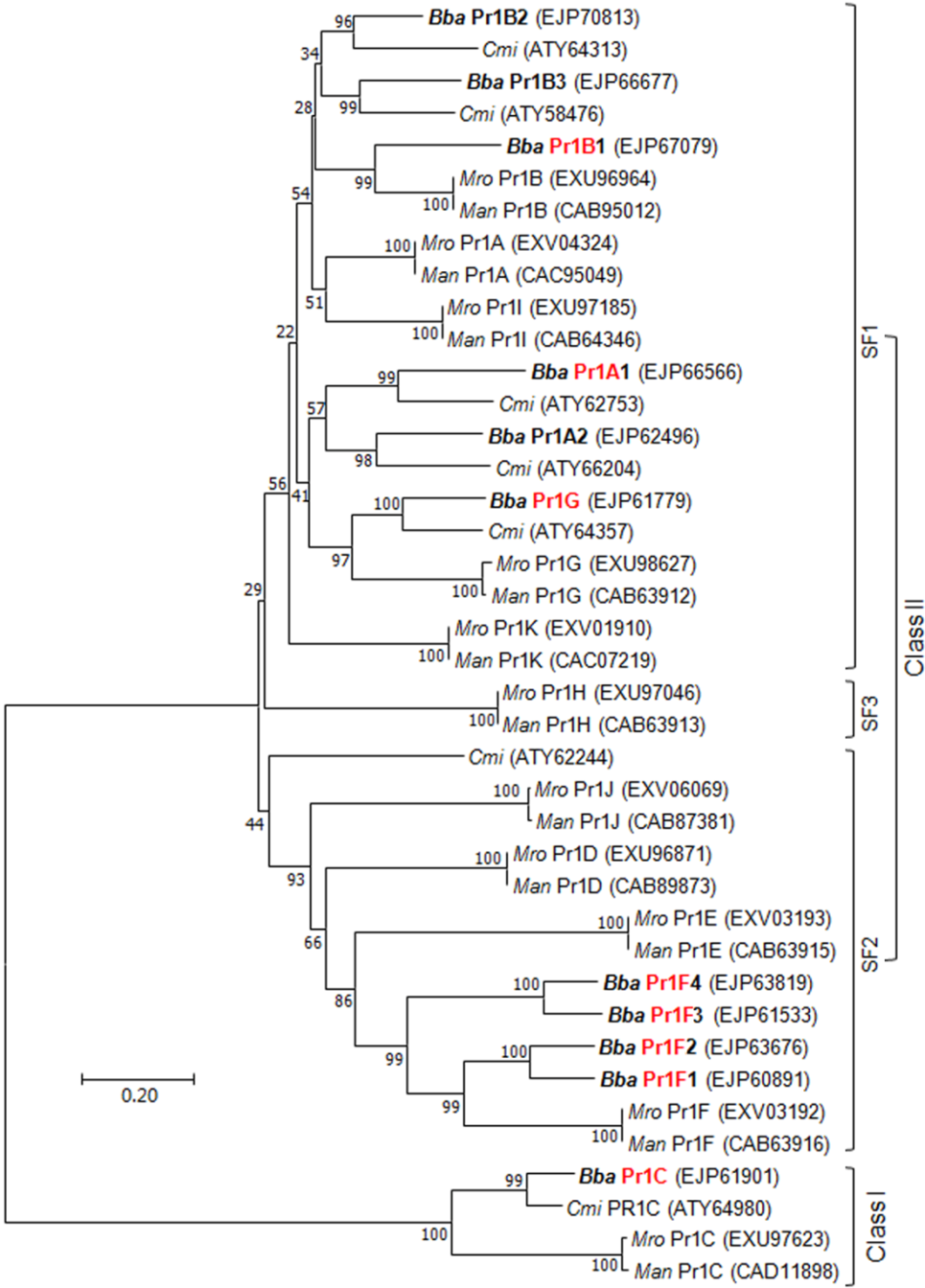


**Fig. S1.** Phylogenetic relationships of all Pr1 proteases early named in *Metarhizium anisopliae* (*Man*) complex and those later annotated in the genomes of representative insect mycopathogens. *Mro*, *Metarhizium robertsii*. *Bba*, *Beauveria bassiana.* *Cmi*, *Cordyceps militaris*. The bootstrap values of 1000 replications are given at nodes. Scale: branch length proportional to genetic distance assessed with the neighbor-joining method in MEGA7 software at http://www.megasoftware.net. Each abbreviated fungal name is followed by a Pr1 protease early named or later annotated (shown in red for *Bba*) and its NCBI accession code in parentheses.


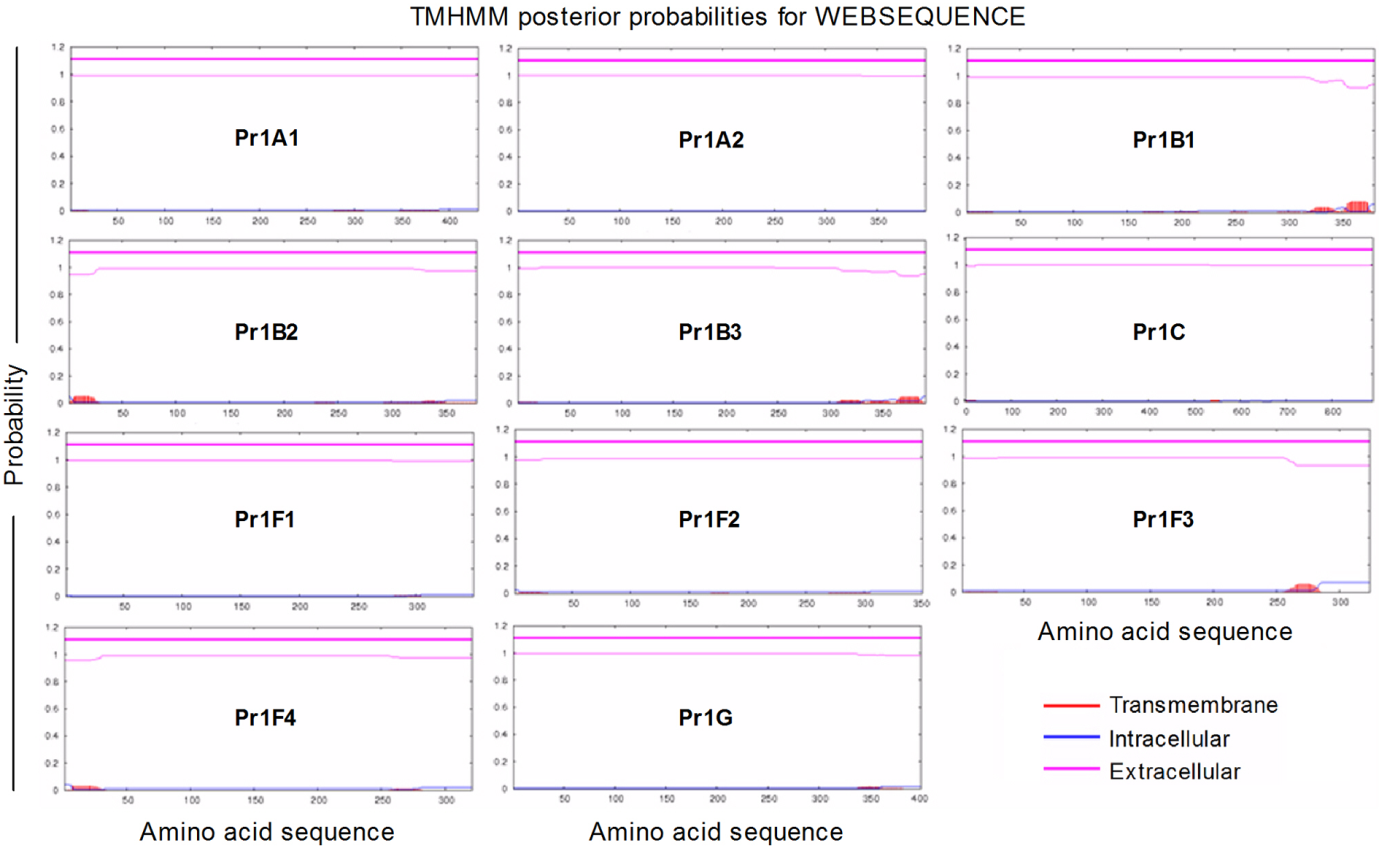


**Fig. S2.** The probabilities predicted for extracellular, intracellular and transmembrance activities of 11 *B. bassiana* Pr1 proteases at http://www.cbs.dtu.dk/services/TMHMM/. Note the high probability of each Pr1 protease acting as an extracellular enzyme in *B. bassiana*.


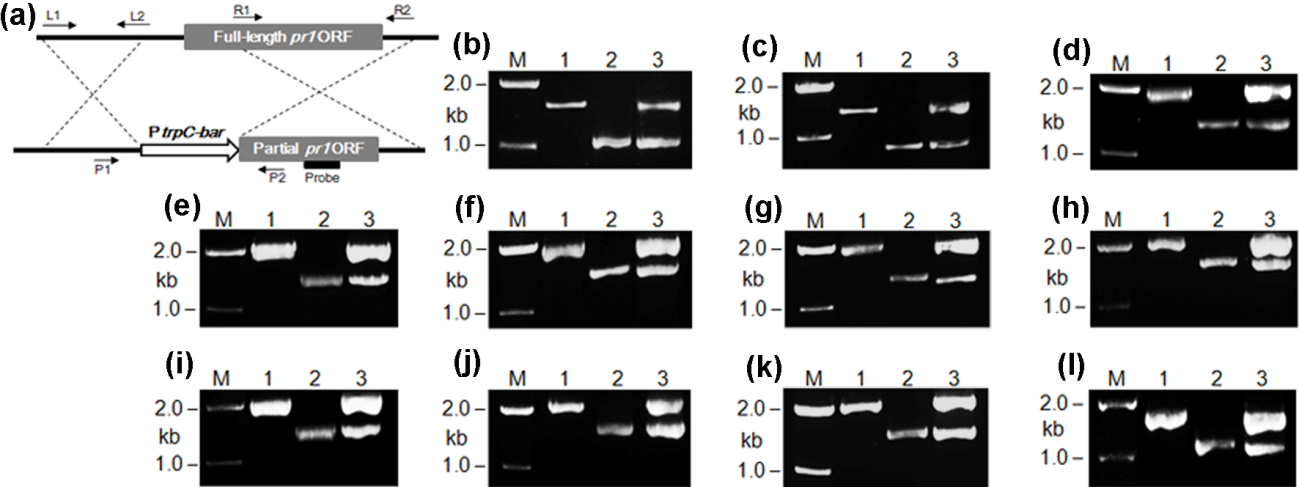


**Fig. S3.** Generation and identification of *B. bassiana* *pr1* mutants. (**a**) Schematic diagram for the deletion strategy of each *pr1* gene. (**b–l**) Detecting *pr1A1*, *pr1A2*, *pr1B1*, *pr1B2*, *pr1B3*, *pr1C*, *pr1F1*, *pr1F2*, *pr1F3*, *Pr1F4* and pr1G from the genomic DNAs of wild-type (lane 1) and corresponding deletion (lane 2) and complemented mutants (lane 3) by PCR with paired primers (Table S1) respectively. M, molecular marker.


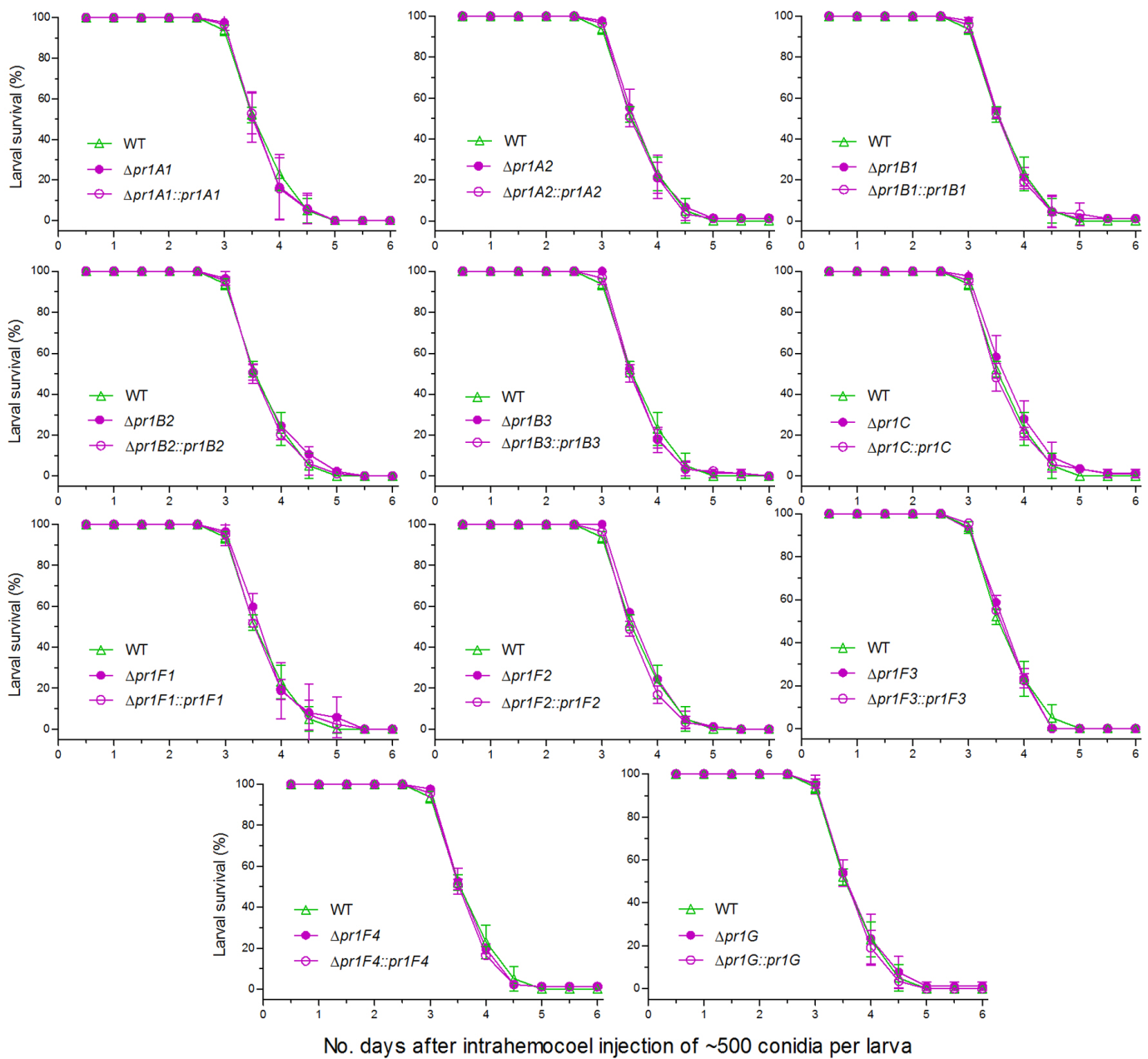


**Fig. S4.** Survival trends of *G. mellonella* larvae after intrahaemocoel injection of ~500 conidia per larva for cuticle-bypassing infection. Error bars: SD from three replicates (three groups of larvae injected per strain, ~35 larvae per group). Note that survival trends are identical for each Δ*pr1* mutant and its control (wild- type and complemented) strains.


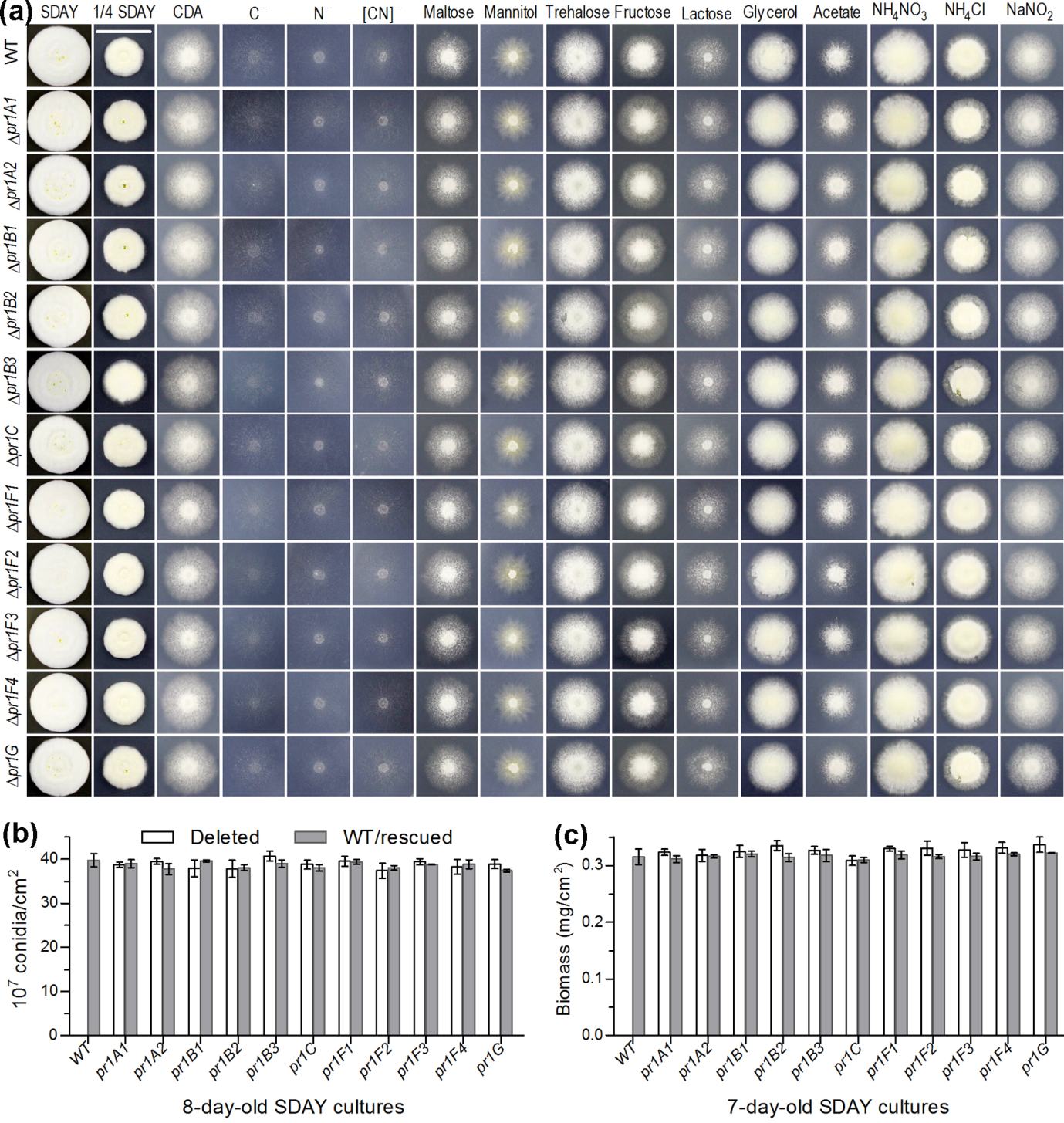


**Fig. S5.** Impacts of singular *pr1* deletions on hyphal growth and aerial conidiation of *B. bassiana*. (**a**) Images of fungal colonies (scale = 2 cm) grown at 25°C for 8 days on the plates of rich SDAY, 1/4 SDAY, CDA and CDAs amended with different carbon/nitrogen sources or in the absence of carbon source (C^−^), nitrogen source (N^−^) or both ([CN]^−^). Each colony was initiated by spotting 1 μl of a 10^6^ conidia/ml suspension. (**b** and **c**) Conidial yields and biomass levels quantified from the 8- and 7-day-old SDAY cultures respectively. Each plate culture was initiated by spreading 1 μl of a 10^6^ conidia/ml suspension and incubated at the optimal regime of 25°C in a light/dark cycle of 12:12 h. There is no significant difference in conidial yield or biomass level between each Δ*pr1* mutant and its control strains (Tukey's HSD, *P* > 0.05). Error bars: SD from three replicates.


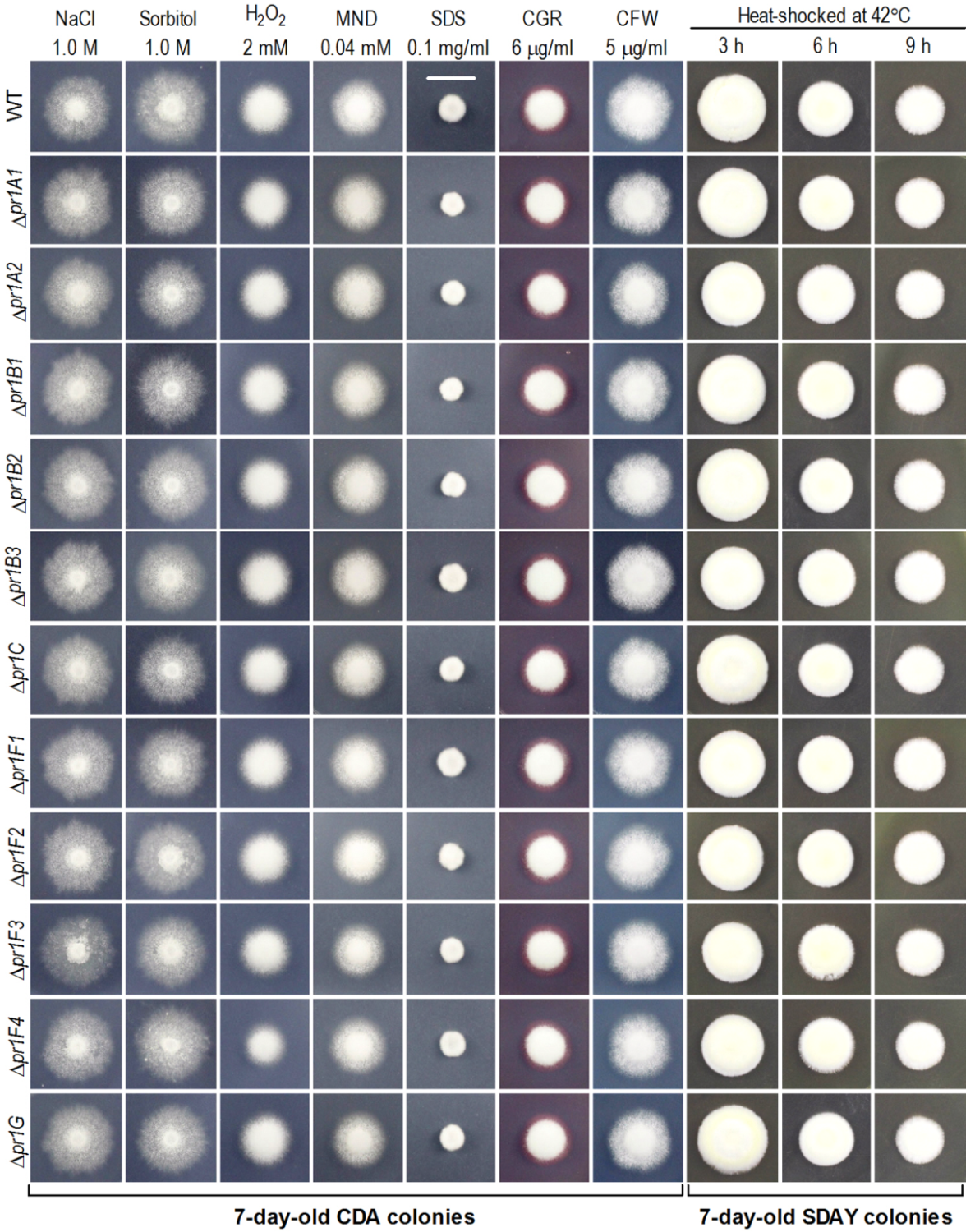


**Fig. S6.** Impacts of singular *pr1* deletions on *B. bassiana* responses to different types of stress cues during 7-day colony growth at optimal 25°C. All colonies were initiated by spotting 1 μl aliquots of a 10^6^ conidia/ml suspension on the plates of CDA supplemented with indicated concentrations of NaCl or sorbitol for osmotic stress, menadione (MND) or H_2_O_2_ for oxidative stress, and sodium dodecyl sulfate (SDS), Congo red (CGR) or calcofluor white (CFW) for cell wall perturbing stress respectively. All SDAY colonies initiated as above were incubated 2.5 days at 25°C, exposed 3-, 6- or 9-h to a 42°C heat shock, and incubated again at 25°C for 4.5-day growth recovery. Colony images (scale = 1 cm) at the end of a 7-day incubation indicate identical responses of all Δ*pr1* mutants and their control strains to each of tested stress cues (diameter measurements not shown).

**Table S1.** Bioinformatic analysis of all Pr1 family proteases found in the genome of *Beauveria bassiana* ARSEF 2860.

| Pr1  family  member | Genomic  tag locus | Gene  sequence  (bp) | No.  introns | cDNA sequence  (bp) | Mol.  Size  (kD) | *p*I | Sequence identity (%) to one another | | | | | | | | | | |
| --- | --- | --- | --- | --- | --- | --- | --- | --- | --- | --- | --- | --- | --- | --- | --- | --- | --- |
|  |  |  |  |  |  |  | Pr1A1 | Pr1A2 | Pr1B1 | Pr1B2 | Pr1B3 | Pr1C | Pr1F1 | Pr1F2 | Pr1F3 | Pr1F4 | Pr1G |
| Pr1A1 | BBA_04506 | 1375 | 1 | 1296 | 46.00 | 6.56 | 100 |  |  |  |  |  |  |  |  |  |  |
| Pr1A2 | BBA_08580 | 1439 | 3 | 1194 | 41.33 | 5.50 | 45 | 100 |  |  |  |  |  |  |  |  |  |
| Pr1B1 | BBA_03653 | 1356 | 3 | 1143 | 39.03 | 8.29 | 42 | 45 | 100 |  |  |  |  |  |  |  |  |
| Pr1B2 | BBA_00443 | 1341 | 3 | 1140 | 38.90 | 7.67 | 44 | 49 | 56 | 100 |  |  |  |  |  |  |  |
| Pr1B3 | BBA_04617 | 1386 | 2 | 1179 | 40.16 | 5.44 | 43 | 48 | 52 | 57 | 100 |  |  |  |  |  |  |
| Pr1C | BBA_09153 | 2841 | 2 | 2670 | 93.71 | 5.83 | 41 | 28 | 27 | 26 | 44 | 100 |  |  |  |  |  |
| Pr1F1 | BBA_10157 | 1050 | 0 | 1050 | 37.84 | 8.81 | 35 | 32 | 35 | 30 | 34 | 33 | 100 |  |  |  |  |
| Pr1F2 | BBA_07320 | 1053 | 0 | 1053 | 37.67 | 8.71 | 36 | 32 | 35 | 31 | 35 | 36 | 78 | 100 |  |  |  |
| Pr1F3 | BBA_09501 | 975 | 0 | 975 | 34.77 | 9.44 | 41 | 32 | 37 | 33 | 34 | 32 | 45 | 45 | 100 |  |  |
| Pr1F4 | BBA_07143 | 966 | 0 | 966 | 34.53 | 9.61 | 39 | 36 | 38 | 34 | 37 | 33 | 46 | 45 | 82 | 100 |  |
| Pr1G | BBA_09270 | 1478 | 3 | 1203 | 42.02 | 6.37 | 46 | 43 | 47 | 39 | 49 | 46 | 37 | 35 | 36 | 37 | 100 |

**Table S2.** Paired primers used for manipulation of eleven *pr1* genes in *B. bassiana*.

| Primers | Paired sequences (5′−3′) ^a^ | Purpose ^b^ |
| --- | --- | --- |
| Pr1A1up-F/R | CCCGGCGCGCCGAATTCCCGGGGCAAGTTGGGACGGAAAAA / CGACCCATGGGAGC TCAAGCTTCGATGAATGAGACGGGTGA | Cloning *pr1A1* 5′ fragment (1331 bp) for deletion |
| Pr1A1dn-F/R | ATTTGATGACCCATGGCTCGAGGGGGAGACGGTGGAAAAGT / GGTGGTGGTGGCTA GCGTTAACTATCAGCACAGCGTGGGGT | Cloning *pr1A1* 3′ fragment (1499 bp) for deletion |
| Pr1A1fl-F/R | TTGGGCCCGGCGCGCCGAATTCGACTGGAACCGAGTATATATGGA / TTGGCTGCAGG TCGACGGATCCCGTATCAGCACAGCGTGG | Cloning full-length *pr1A1* 4003 bp) for complementation |
| Pr1A2up-F/R | CCCGGCGCGCCGAATTCCCGGGGTTTTGTGTGGTAGATGGGTG / TTGACCATGGGAGCT CAAGCTTACATTGCGAGTTGATGGGAT | Cloning *pr1A2* 5′ fragment (1579 bp) for deletion |
| Pr1A2dn-F/R | GACCCATGGCTCGAGTCTAGACAGCACCCTGATGTAAGTCG / GGTGGTGGTGGCTAGC GTTAACTTCTTGAGCGTCTGGAACC | Cloning *pr1A2* 3′ fragment (1775 bp) for deletion |
| Pr1A2fl-F/R | TTGGGCCCGGCGCGCCGAATTCTCCACAGTGTTTCTTATGGCTCA / TTGGCTGCAGGT CGACGGATCCTGTCTGAGAGGATGGGACTGACT | Cloning full-length *pr1A2* (4238 bp) for complementation |
| Pr1B1up-F/R | TGGGCCCGGCGCGCCGAATTCCGTCAACCTCAGTCACCAAT / CGACCCATGGGAGCTCA AGCTTTCTCATCTCACCACTCCAGC | Cloning *pr1B1* 5′ fragment (1745 bp) for deletion |
| Pr1B1dn-F/R | ATTTGATGACCCATGGCTCGAGTTCTAACAGACTCGACGCAGG / GGTGGTGGTGGCTAG CGTTAACAGCAAGACCAATCACTAATCCA | Cloning *pr1B1* 3′ fragment (1422 bp) for deletion |
| Pr1B1fl-F/R | TTGGGCCCGGCGCGCCGAATTCAACTGAACCTACTACCCGATATCA / TTGGCTGCAGGTC GACGGATCCCCTCCTTTTAGCAAGACCAAT | Cloning full-length *pr1B1* (4036 bp) for complementation |
| Pr1B2up-F/R | TTGGGCCCGGCGCGCCGAATTCTGGTGACGACGGAGTGTGGT / TTGGCTGCAGGTCGAC GGATCCTTGCTTGCTGGATGAAGTAGAAG | Cloning *pr1B2* 5′ fragment (1092 bp) for deletion |
| Pr1B2dn-F/R | GACCCATGGCTCGAGTCTAGACGAGTCCATCGAGCAGGA / GGTGGTGGTGGCTAGCGT TAACGAACCAATCTAAGTCGTAAATAACA | Cloning *pr1B2* 3′ fragment (1383 bp) for deletion |
| Pr1B2fl-F/R | TTGGGCCCGGCGCGCCGAATTCAACATCAAAGTAACGAGACAGC / TTGGCTGCAGGTC GACGGATCCCCTTTTTGTTGTTGACTCTGAA | Cloning full-length *pr1B2* (4073 bp) for complementation |
| Pr1B3up-F/R | TGGGCCCGGCGCGCCGAATTCCACCACTATTCTCCCTGTCCTT / TTGGCTGCAGGTCGAC GGATCCCTTTCAGTCGCCTTCATCTTCT | Cloning *pr1B3* 5′ fragment (1581 bp) for deletion |
| Pr1B3dn-F/R | TGACCCATGGCTCGAGTCTAGATCCAAACTTTCTCAGCCCC / GGTGGTGGTGGCTAGCG TTAACATGCCTCTGTGTATCCTCGC | Cloning *pr1B3* 3 fragment (1566 bp) for deletion |
| Pr1B3fl-F/R | TTGGGCCCGGCGCGCCGAATTCCAGAAGAGAGGCTTGTTGAGGC / TTGGCTGCAGGTC GACGGATCCCGTTTACGGCGGTTTCGAC | Cloning full-length *pr1B3* (4119 bp) for complementation |
| Pr1Cup-F/R | CCCGGCGCGCCGAATTCCCGGGTTTGTGGGGGAACTGAGATG / TTGGCTGCAGGTCGA CGGATCCCGAGAATGCGGGTGCTTG | Cloning *pr1C* 5′ fragment (1623 bp) for deletion |
| Pr1Cdn-F/R | TGACCCATGGCTCGAGTCTAGAACGGCGGTTGTTGGTCAG / GGTGGTGGTGGCTAGC GTTAACGAGCAGGGGCATTGTTGT | Cloning *pr1C* 3′ fragment (1421 bp) for deletion |
| Pr1Cfl-F/R | TTGGGCCCGGCGCGCCGAATTCAAAAAAACAAAGTCAGAGAGCAT / TTGGCTGCAGG TCGACGGATCCACCTTCAGCCGATTCTTCAG | Cloning full-length *pr1C* (5221 bp) for complementation |
| Pr1F1up-F/R | CCGGCGCGCCGAATTCCCGGGATTGCGTAGCCTTCATAGTGTG / TGGCTGCAGGTCGAC GGATCCGAGCACAGAGGGGATTTGGT | Cloning *pr1F1* 5′ fragment (1293 bp) for deletion |
| Pr1F1dn-F/R | GACCCATGGCTCGAGTCTAGACGTGCTGAGAATCTTTGGACA / GGTGGCTAGCGTTAAC ACTAGTGGCGTGCTATCATTTTCGG | Cloning *pr1F1* 3′ fragment (1341 bp) for deletion |
| Pr1F1fl-F/R | TTGGGCCCGGCGCGCCGAATTCTATGCTCATTTTTCAAGTCTGCC / TTGGCTGCAGGTCG ACGGATCCAAAGGTCAAGTTGGGTCTATGTCA | Cloning full-length *pr1F1* (3999 bp) for complementation |
| Pr1F2up-F/R | TGGGCCCGGCGCGCCGAATTCAACGACGCTAATGAAGGGAA / TGCAGGTCGACGGATC CCCGGGGATAAAATTACGGACCAGGCAC | Cloning *pr1F2* 5′ fragment (1545 bp) for deletion |
| Pr1F2dn-F/R | AGTCTAGAAGATCTGACTAGTCAAGGAGTTTGAGGGTCGTG / GGTGGTGGTGGCTAGC GTTAACGTCAAGTTTGGGTCATCGTA | Cloning *pr1F2* 3′ fragment (1220 bp) for deletion |
| Pr1F2fl-F/R | TTGGGCCCGGCGCGCCGAATTCTATTGGAGGATTCAGATTCTGCT / TTGGCTGCAGGTC GACGGATCCCGGTGGAGGCGTACAGTAATT | Cloning full-length *pr1F2* (4066 bp) for complementation |
| Pr1F3up-F/R | CCGGCGCGCCGAATTCCCGGGTCAAGTGTCTCGGGAGTAAATA / TGGCTGCAGGTCGAC GGATCCGCAAAGTAATCAATCAGTTCAGG | Cloning *pr1F3* 5′ fragment (1552 bp) for deletion |
| Pr1F3dn-F/R | GACCCATGGCTCGAGTCTAGACCTGTGGACGGCTGATCTG / GTGGCTAGCGTTAACACT AGTCCCCTATTGCGGGTTACTTT | Cloning *pr1F3* 3′ fragment (1416 bp) for deletion |
| Pr1F3fl-F/R | TTGGGCCCGGCGCGCCGAATTCTTCTCAGTCAGGGGAACCAC / TTGGCTGCAGGTCGAC GGATCCTTGATAGAGGAGCAAAAGAAGC | Cloning full-length *pr1F3* (3931 bp) for complementation |
| Pr1F4up-F/R | TGGGCCCGGCGCGCCGAATTCTGTAGCCTTGGAATCCGTAGA / TGGCTGCAGGTCGACG GATCCACGAACCACAAATGTCTCTGC | Cloning *pr1F4* 5′ fragment (1431 bp) for deletion |

**Table S2** (continued)

| Primers | Paired sequences (5′−3′) ^a^ | Purpose ^b^ |
| --- | --- | --- |
| Pr1F4dn-F/R | GACCCATGGCTCGAGTCTAGACGAGCCCAACTACGACGATAC / GGTGGTGGTGGCTAGC GTTAACGGACTATTCGTTAGGCGACCAC | Cloning *pr1F4* 3′ fragment (1021 bp) for deletion |
| Pr1F4fl-F/R | TTGGGCCCGGCGCGCCGAATTCGGGAAGGTCGTCAAAAATGGT / TTGGCTGCAGGTCGA CGGATCCCGTCGGATGTATTATCGTAGGC | Cloning full-length *pr1F4* (3689 bp) for complementation |
| Pr1Gup-F/R | TGGGCCCGGCGCGCCGAATTCAAGCCTGGAGGAAGAAAAGA / TGGCTGCAGGTCGACG GATCCAGGAGGACGAGGATTGTGTT | Cloning *pr1G* 5′ fragment (1509 bp) for deletion |
| Pr1Gdn-F/R | AGTCTAGAAGATCTGACTAGTCCGTCTACTTGGGGTCTCG / GGTGGTGGTGGCTAGCGT TAACGCATACCGTGGTGAAAGATT | Cloning *pr1G* 3′ fragment (1590 bp) for deletion |
| Pr1Gfl-F/R | TTGGGCCCGGCGCGCCGAATTCTTTGATGCGTGATGACACTATATC / TTGGCTGCAGGTC GACGGATCCATGACCTTTCGACTACGCTCTATA | Cloning full-length *pr1G* (4312 bp) for complementation |
| pPr1A1-F/R | CAACTCCCATTTGGTTCACTCA / GATGTAGACGCAGACGCCC | PCR detecting *pr1A1* |
| pPr1A2-F/R | GTCTGAGGGAAGTGAGTGAGGC / CGTAGTTGTACTGGTTGCTGTTGG | PCR detecting *pr1A2* |
| pPr1B1-F/R | CAATGCTTCTTGGCTTTATGAG / CCGAGAGCGTTTGCTGTTTA | PCR detecting *pr1B1* |
| pPr1B2-F/R | CTCCGGTACGACTGCCTACT / GTTTTATTTGAACGGCGTGA | PCR detecting *pr1B2* |
| pPr1B3-F/R | TGACGGTGAGTGATTGAACAT / TAGACGCTGGCTTTTTTGG | PCR detecting *pr1B3* |
| pPr1C -F/R | AACTACGCTGTAAGCCTCCTC / GCCTCTTCTTTTCTTTTCCTCT | PCR detecting *pr1C* |
| pPr1F1-F/R | ATTGTTATCAAGTCAGACCCAGA / CGCCTAACACTCATCGTCCT | PCR detecting *pr1F1* |
| pPr1F2-F/R | GCATCCGATACCCCACAACC / CGCCCTGTCAATGATGGTGT | PCR detecting *pr1F2* |
| pPr1F3-F/R | CGTAGTTTACTCTATCTAATCGGG / AACGAAACCGCTGACCTAT | PCR detecting *pr1F3* |
| pPr1F4-F/R | AACCAGGGAGATAGGCATAAC / TGGATAGCTGACAGTAGATTTGAT | PCR detecting *pr1F4* |
| pPr1G -F/R | TGATCGACTGCGACAGACTAG / CACTGTTGATGAATTACGGAAAC | PCR detecting *pr1G* |
| qPr1A1-F/R | CGACAGGTACATCATCAA/ GAACTTGTTCTTGTAGCG | qPCR detecting *pr1A1* |
| qPr1A2-F/R | CATTATCGCCGACAAGT / CCTAGAAAGACACCTTCC | qPCR detecting *pr1A2* |
| qPr1B1-F/R | AGTGTAGAGTACATTGAG / AGAGCTGTCATAGGTAT | qPCR detecting *pr1B1* |
| qPr1B2-F/R | GGCAAGTACATTGTCAAG / AAGTTGTTCTTGGTTGAG | qPCR detecting *pr1B2* |
| qPr1B3-F/R | CAGCTTCTCCAACTACGG/ CCATAGAGGTGCCAGAT | qPCR detecting *pr1B3* |
| qPr1C-F/R | CAAGATTGTCCTCATCC / GGCATTGTTGTAGATGATG | qPCR detecting *pr1C* |
| qPr1F1-F/R | TACTACTATCAATCGTGG / GTCTTCAAAGTCGTTCTT | qPCR detecting *pr1F1* |
| qPr1F2-F/R | AAGTATGCCATTGTGAAT / AAACTCCTTGTGTGTAAT | qPCR detecting *pr1F2* |
| qPr1F3-F/R | CATATTGTATCCGTCAAG / GCCTAACGAGATATTAATG | qPCR detecting *pr1F3* |
| qPr1F4-F/R | AACATGGATCAAGATTGG / TCATCAACTGAGACTTCT | qPCR detecting *pr1F4* |
| qPr1G-F/R | AATATGTGGAGCAGAAT / AAGAGTCGTAAAGGTAAT | qPCR detecting *pr1G* |
| qActin-F/R | GGCAACATTGTCATGTCTGG / TTTGCTGGAAGGTGGATAGG | qPCR detecting *β-actin* gene |

a. Underlined regions denote the introduced cleavage sites of restriction enzymes for homogeneous recombination of 5′ and 3′ fragments to delete *pr1A1* (*Sma*I/*Hin*dIII and *Xho*I/*Hpa*I), *pr1A2* (*Sma*I/*Hin*dIII and *Xba*I/*Hpa*I), *pr1B1* (*Eco*RI/*Hin*dIII and *Xho*I/*Hpa*I), *pr1B2/B3/F4* (*EcoR*I/*BamH*I and *Xba*I/*Hpa*I), *pr1C* (*Sma*I/*BamH*I and *Xba*I/*Hpa*I), *pr1F2* (*Eco*RI/*Sma*I and *Xba*I/*Hpa*I), *pr1F1/F3* (*Xma*I/*BamH*I and *Xba*I/*Spe*I) and *pr1G* (*EcoR*I/*BamH*I and *Spe*I/*Hpa*I) respectively or the gateway exchange fragments to rescue each gene in an identified deletion mutant.

b. PCR detection aimed at the fragments of deleted *pr1A1* (1493 bp) vs. WT (1077 bp), deleted *pr1A2* (1400 bp) vs. WT (906 bp), deleted *pr1B1* (1858 bp) vs. WT (1360 bp), deleted *pr1B2* (1996 bp) vs. WT (1446 bp), deleted *pr1B3* (1811 bp) vs. WT (1454 bp), deleted *pr1C* (1828 bp) vs. WT (1297 bp), deleted *pr1F1* (1975 bp) vs. WT (1550 bp), deleted *pr1F2* (2142 bp) vs WT (1534 bp), deleted *pr1F3* (2027 bp) vs. WT (1537 bp), deleted *pr1F4* (1920 bp) vs. WT (1520 bp), and deleted *pr1G* (1600 bp) vs. WT (1145 bp), respectively.
